# A neuro-computational account of procrastination behavior

**DOI:** 10.1038/s41467-022-33119-w

**Published:** 2022-09-26

**Authors:** Raphaël Le Bouc, Mathias Pessiglione

**Affiliations:** 1 Motivation, Brain and Behavior (MBB) Lab, Paris Brain Institute (ICM), Sorbonne University, Inserm, CNRS, Pitié-Salpêtrière hospital, Paris, France; 2 Department of Neurology, Pitié-Salpêtrière hospital, Sorbonne University, Assistance Publique – Hôpitaux de Paris (APHP), Paris, France

**Keywords:** Decision, Reward, Motivation, Computational neuroscience, Human behaviour

## Abstract

Humans procrastinate despite being aware of potential adverse consequences. Yet, the neuro-computational mechanisms underlying procrastination remain poorly understood. Here, we use fMRI during intertemporal choice to inform a computational model that predicts procrastination behavior in independent tests. Procrastination is assessed in the laboratory as the preference for performing an effortful task on the next day as opposed to immediately, and at home as the delay taken in returning completed administrative forms. These procrastination behaviors are respectively modeled as unitary and repeated decisions to postpone a task until the next time step, based on a net expected value that integrates reward and effort attributes, both discounted with delay. The key feature that is associated with procrastination behavior across individuals (both in-lab and at-home) is the extent to which the expected effort cost (signaled by the dorsomedial prefrontal cortex) is attenuated by the delay before task completion. Thus, procrastination might stem from a cognitive bias that would make doing a task later (compared to now) appear as much less effortful but not much less rewarding.

## Abstract (web summary)

Most humans procrastinate to some extent, despite adverse consequences. Here, the authors show that how much an individual procrastinates, both in the lab and at home, relates to brain signals that reflect temporal discounting of effort cost.

## Introduction

Almost all humans procrastinate to some extent, either on filling tax returns, paying bills, saving for retirement, or quitting addictive behaviors like smoking or gambling. People do so although knowing about potential adverse consequences, such as financial difficulty^1,2^ or health damage^3^. Despite its high prevalence, affecting ~70% of students^4^ and up to 20% of adults^5^, and its major economic or health consequences, the mechanisms leading to procrastination remain poorly understood.

Procrastination is considered a stable trait-like behavior^6^, with significant heritability demonstrated by twin studies^7^. However, the causal pathways through which genes could shape the brain architecture so as to produce procrastination behavior are not elucidated. Neuroimaging studies have not gone beyond correlations between procrastination scores on self-report questionnaires and brain anatomy, resting-state activity^8–11^, or task-related activity^12–14^. This questionnaire-based approach offers no mechanistic insight into the emergence of procrastination, which would require an operational definition at the cognitive level.

As suggested by its etymology (crastinus is a Latin word for tomorrow), the common meaning of procrastination is to postpone duties from one day to the next. This definition has been refined by psychologists as the unnecessary but voluntary delaying of task completion (either requested or intended) despite potential harmful outcomes^4,6,15,16^. For ancient philosophers such as Aristotle, procrastination is a prototypical case of akrasia, which designates a lack of self-control leading to act against one’s best judgment. This perspective is still present in the psychological literature on procrastination, which may be considered as resulting from self-regulation failure or ‘weakness of the will’^6,17,18^.

In the framework of neoclassical economic theory, procrastination would be considered irrational, because it prevents maximizing utility in the long run, even when the right course of action is clearly identified. This seemingly irrational behavior has given rise to the development of alternative economic models that would preserve the principle of utility maximization.

An early economic account of procrastination^1^ emphasized the importance of costs, meaning the effort and time associated with task completion, which should be traded against the remote benefits associated with the outcome of task completion. Procrastination would stem from present costs (if the task is done now) being perceived in a much more vivid manner than distant costs (if the task is done later)^1^. Thus, in this model, the present bias leading to procrastination is captured by a vividness parameter that amplifies the cost of immediate actions^1^. A variant of this model focuses on the opportunity cost of time, defined as the value of what is forgone when implementing a particular action^6^. In this variant, procrastination would result from the distant benefit of task completion being surpassed by the opportunity cost, i.e. the lost benefit of what could be enjoyed now. Thus, in both cases, a present bias would explain procrastination, either because completing the task now would be perceived as particularly aversive, or because enjoying now an alternative activity would seem particularly attractive.

To be complete, the model should explain not only why people procrastinate (why they postpone a task) but also why they stop procrastinating at some point (why they finally do it). Further variants of the procrastination model benefited from the development of economic decision theory in which expected utility is progressively discounted with the passage of time^19–21^, as opposed to a mere present bias. The core idea was to apply the same temporal discounting function to both costs and benefits. Functions that would diminish expected utility by some percentage every day would not account for procrastination, since they predict stable preferences over time. However, empirical work suggested that human choices are better accounted for by inconsistent temporal discounting models, using for instance hyperbolic functions^21^, in which the daily discount decreases over time^19,20^. These models allow for preference reversal, i.e. the inversion of the ranking between options when their outcomes get closer in time. Applied to the case of procrastination, preference reversal would mean that work may appear less valuable than leisure when deadlines are distant, but may nonetheless be favored when deadlines are closer^6^. This idea has been incorporated in recent psychological investigations^22^, following on the temporal motivation theory^6,23^, which borrows from economic decision models the notions of expected utility, temporal discounting and cost/benefit trade-off.

Thus, both economic and psychological accounts of procrastination build on assumptions about temporal discounting functions and share some limitations. First, these models only account for situations involving remote benefits, or at least a lag between task completion and reward delivery, although procrastination has also been observed with tasks that yield immediate benefits, such as redeeming gift vouchers^24^. Second, these models implicitly or explicitly consider that avoiding a cost is equivalent to receiving a benefit, the timing of benefits and the timing of costs being considered as just flip sides of the same phenomenon^25^. This makes procrastination a synonym of impulsivity—the preference for smaller sooner over larger later benefits—although these two traits have been shown to be dissociable^7^. Moreover, recent studies have shown that reward value and effort cost are processed by distinct neural circuits^26,27^, which may suggest separate processes for the temporal discounting of costs and benefits. Third, these models are static, in the sense that the decision about completion time is made once and for all, which is at odds with the psychological intuition that procrastination entails iterative decisions (to defer again and again).

Here, we suggest a framework that may overcome these limitations, by combining the seminal intuitions that procrastination relates to both effort perception and temporal discounting, resulting in the following assumptions: (1) procrastination stems from choice between options that integrate costs and benefits both estimated at different time points (now and later), (2) a unitary decision to procrastinate reflects a steeper temporal discounting for effort than for reward^28^, and (3) recurrent procrastination arises from iterative decisions repeated over time. From this set of assumptions, we derive the predictions that procrastinators would discount efforts with time more steeply than non-procrastinators, that these time preferences would be associated with neural activity reflecting temporal discounting of effort, and that dynamic models would outperform static models in predicting real-life procrastination. To test these predictions, we measure temporal discounting rates for reward and effort in intertemporal choice tasks and identify their neural signature using fMRI. We find that temporal discounting of effort cost, either inferred from choice behavior or from brain activity, could account for procrastination behavior, observed both in the lab, as a preference for postponing an effortful task until the next day, and at home, as a delay in returning completed administrative forms.

## Results

**Fig. 1:**
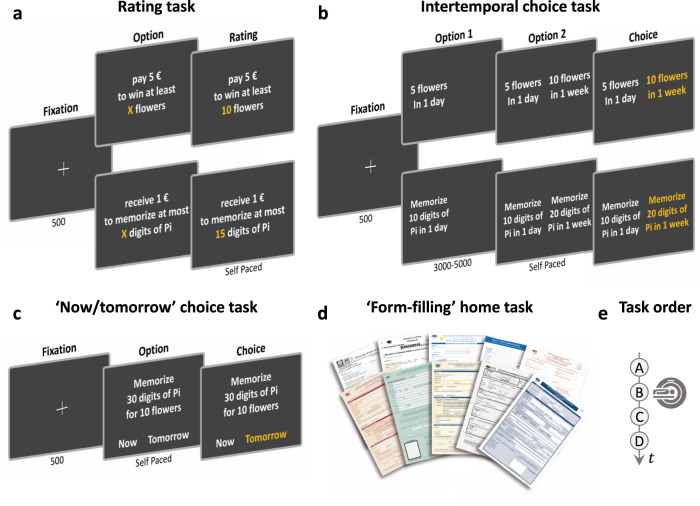
Behavioral tasks. Successive screens displayed in one trial are shown from left to right, with durations in ms. **a** Rating task. For each reward, effort and punishment (not shown), participants indicated on a keyboard the quantity that had the same subjective benefit (or subjective cost) than earning (or loosing) 1€ and 5€. **b** Intertemporal choice task. Participants first observed the two options shown successively and then indicated their preference by pressing one of two buttons with the left or right hand. The presentation order of sooner and later options was counterbalanced across trials. The task was divided into blocks of intertemporal choices between two rewards, two efforts, or two punishments (not shown). **c** ‘Now/tomorrow’ choice task. Participants were presented with an option combining reward and effort items. Then they indicated whether they preferred to exert the effort “Now”, and obtain the reward immediately, or “Tomorrow”, and obtain the reward the next day. The side of presentation of the “Now” and “Tomorrow” options was counterbalanced across trials. **d** ‘Form-filling’ home task. Participants were given 10 administrative forms, such as a passport renewal form. They had to fill in the forms and send a numeric copy via email within a time limit of 30 days, in order to receive their financial compensation for participating in the study. They were told that no compensation would be transferred after the deadline. **e** Experimental schedule. Tasks were performed in the alphabetic order. Only the intertemporal choice task was performed in the MRI scanner.

A total of 51 healthy adult volunteers performed a series of behavioral tasks: first, rating tasks (Fig. 1a), to collect subjective effort costs and reward values that participants would assign to each task and outcome included in our set of items; second, intertemporal choice tasks (Fig. 1b), to elicit computational and neural markers of temporal discounting for both the effort and reward domains; and third, tasks measuring the tendency to procrastinate, both in the lab (Fig. 1c) and at home (Fig. 1d), to assess the predictive validity of these markers. All tasks were performed in that order (Fig. 1e), during a single visit to the lab, fMRI scanning being only applied to the intertemporal choice task. Participants were divided into three cohorts: one performing a pilot version (Exp. 1) of the intertemporal choice task (*n* = 8), one performing the main version (Exp. 2) of this task (*n* = 16) and one performing the main version (Exp. 2) within the MRI scanner (*n* = 27). Demographic details are provided separately for the three groups in Supplementary Table 1.

### Time preferences

The purpose of the intertemporal choice task was to examine how participants discount costs and benefits with time. In order to infer temporal discount rate, we first needed to know the values that participants would assign to the various reward and effort items presented in the choice task. To this aim, we had participants rate the monetary value of hypothetical reward and effort items on an analog scale (Fig. 1a). Reward items could be either food or goods, and effort items either motor or cognitive tasks. For a given reward item, participants rated how many elements they would claim for a given price (e.g., how many pieces of sushi are worth paying 5€). Reciprocal value ratings were obtained for effort items, by asking participants how much effort they would exert for a given payoff (e.g., how many sit-ups should be done for a fair payment of 5€). Punishment items (either bodily or abstract) were also included in the rating task, as a control for specificity in the second experiment (see below). The question was similar to that asked for effort ratings: participants rated how much punishment they would accept to endure for a given payoff (e.g., how many mild electric shocks should be endured for a fair payment of 5€). Thus, subjective ratings provided monetary values in euros, which represented the equivalent gain for one element of reward items (e.g., the price of one piece of sushi) and the equivalent loss for effort and punishment (e.g., the payoff for one sit-up or one electric shock).

**Fig. 2:**
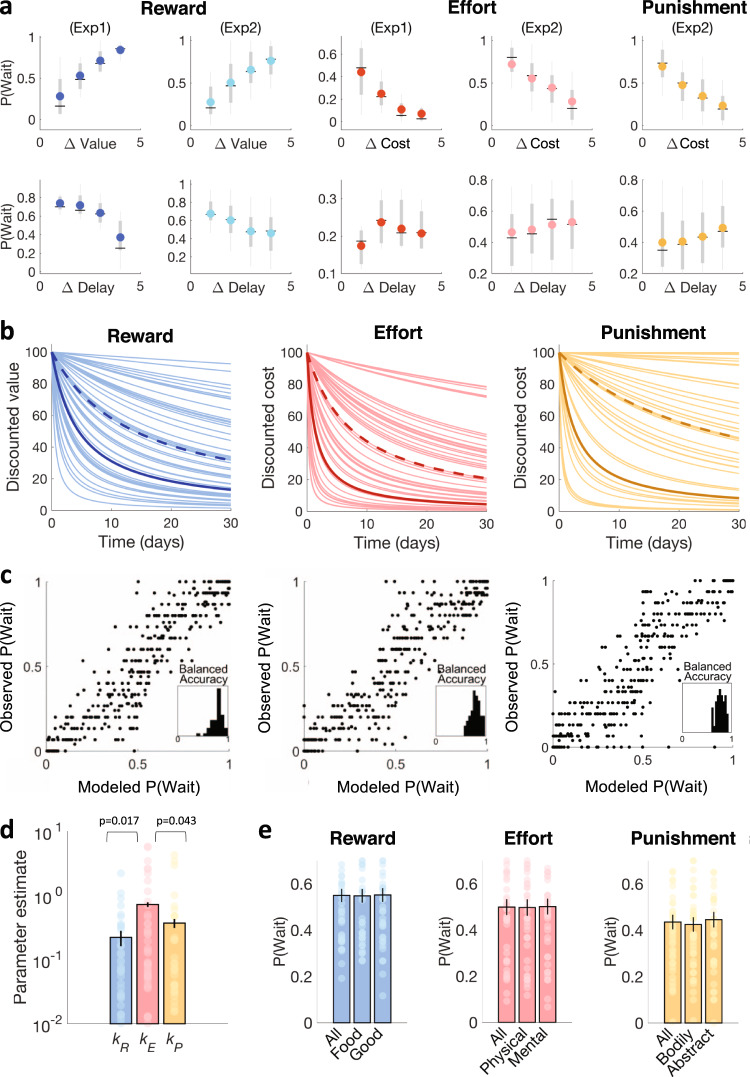
Time preferences. **a** Intertemporal choices. Plots show patient choice rate (preference for the delayed option), as a function of the difference between option values /costs (top row) or option delays (bottom row). Choices were made between two rewards (blue), two efforts (red) or two punishments (yellow). The two options of a choice differed only in delay and value / cost (quantity converted into euros based on subjective ratings). Note that value represents a gain for reward and cost a loss for effort and punishment. In Exp. 1 (*n* = 8 participants), rewards (dark blue) were to be received and efforts (dark red) were to be exerted at the specified date. In Exp. 2 (*n* = 43 participants), rewards (light blue) were to be received at the specified date, but efforts (light red) and punishments (yellow) could be exerted or endured at any time before the specified deadline. On each dot plot, the color mark indicates the mean, the horizonal line indicates the median, the thick whiskers indicate the range from 25th to 75th percentiles, and the thin whiskers indicate the range from 5th to 95th percentiles. **b** Temporal discounting of reward (left), effort (middle), and punishment (right). The plots display individual discount curves for reward (thin blue lines), effort (thin red lines), and punishment (thin orange lines), as well as the population means (bold lines) and medians (dashed lines). **c** Accuracy of model fitting. Plots show correlations between modeled and observed choice probability for delayed rewards, delayed efforts, and delayed punishments. Individual choices were divided into 8 bins of increasing modeled probability; each dot represents modeled and observed choice probability averaged within one bin. The within-subject (trial-by-trial) fit can be assessed with the distribution of balanced accuracy across participants. Balanced accuracy is the average of prediction accuracy calculated separately for the two types of choices (now or later). **d** Temporal discount rates for reward (*k*_*R*_), effort (*k*_*E*_) and punishment (*k*_*P*_). Significance values are based on two-tailed paired *t* tests (*n* = 43). **e** Proportion of patient choices for the different categories of rewards, efforts and punishments. Error bars are inter-subject standard error of the mean (*n* = 43). Source data are provided as a Source Data file.

In the intertemporal choice task, participants indicated their preference between a lower/sooner and a higher/later hypothetical reward, or between a lower/sooner and a higher/later hypothetical effort (Fig. 1b). The two options of a choice were offering a same item already presented in the rating task but with different quantities (e.g., 5 pieces of sushi now vs. 10 pieces of sushi in a week). We changed the framing of delayed efforts between the pilot and test experiments. In a first pilot experiment (*n* = 8), delays indicated the precise dates at which efforts had to be exerted, or at which rewards were to be obtained. We examined the weight of the different choice factors using a logistic regression model (Fig. 2a). Predictably, patient choice frequency in the reward domain was significantly impacted by the relative gain (*β* = 0.44, *t*(7) = 7.74, *p* < 0.001) and negatively impacted by the relative delay (*β* = −0.28, *t*(7) = −7.23, *p* < 0.001). However, in the effort domain, preferences revealed an unexpected pattern: patient choice rate did decrease with the relative cost (*β* = −0.35, *t*(7) = −3.78, *p* = 0.007) but did not show any monotonic effect of delay (*β* = 0.01, *t*(7) = 0.29, *p* = 0.78). The trend was even to expedite the task sooner, denoting a negative time preference. We assumed this reverse preference might have resulted from the framing of delay as the specific date at which effort should be exerted (which might have induced some dread phenomenon).

We therefore conducted a second experiment (*n* = 43) using an intertemporal choice task proposing the same effort options, but with delays now meaning time limits for effort exertion. In Exp. 2, we also introduced punishments in the intertemporal choice task, to test whether temporal discounting of effort would generalize to any aversive dimension. The logistic fit of choice behavior in Exp. 2 showed an opposite pattern in the effort domain compared to the reward domain (Fig. 2a). In the reward domain, patient choice frequency still increased with the relative gain (*β* = 0.34, *t*(42) = 14.67, *p* < 0.001) and decreased with the relative delay between options (*β* = −0.17, *t*(42) = −7.96, *p* < 0.001). However, in the effort domain, the change of framing resulted in the expected pattern, with a negative effect of relative cost (*β* = −0.32, *t*(42) = −11.40, *p* < 0.001) and a positive effect of relative delay (*β* = 0.06, *t*(42) = 3.38, *p* = 0.002), meaning that participants now preferred effort items that were more distant in the future. The same pattern was observed for punishments, with a negative impact of relative loss (*β* = −0.35, *t*(42) = −14.04, *p* < 0.001) and a positive impact of relative delay (*β* = 0.09, *t*(42) = 5.72, *p* < 0.001), denoting again a preference for delaying aversive events.

We then estimated temporal discount rates for reward, effort and punishment by fitting hyperbolic choice models onto choice behavior in Exp. 2 (Fig. 2b). Hyperbolic choice model here means hyperbolic discounting function to generate option value, combined with softmax function to compare option values and generate choice probabilities:

1 $${V}_{A}=\,\frac{{N}_{{Ai}}\,\times \,{R}_{i}}{1+{k}_{R}.{D}_{A}}$$

and

2 $${P}_{A}=\,\frac{1}{1+{e}^{-\theta .({{V}_{A}-V}_{B})}}$$

With *V*_*A*_ the value of option A calculated as the number of elements *N*_*Ai*_ on offer multiplied by the gain equivalent *R*_*i*_ for one unit of reward i (inferred from subjective rating), discounted by the delay *D*_*A*_ multiplied a weight *k*_*R*_ (temporal discount rate for reward). The discounted value of effort and punishment were computed in the same manner, except that gain equivalents were replaced by loss equivalents (similarly inferred from effort and punishment subjective ratings) and that different temporal discount rates (*k*_*E*_ and *k*_*P*_) were used. In addition to temporal discount rate, the softmax included an additional parameter, the inverse temperature *θ*, to adjust for choice stochasticity. We compared this hyperbolic discounting to two classical models of intertemporal choices: a present-bias model (Eq. 3), and a quasi-hyperbolic discounting model (*βδ* model) that includes both a present bias parameter *β* and a constant exponential discount factor *δ* (Eq. 4). Notice that there is a discontinuity in these functions such that, if *D*_*A*_ = 0, *V*_*A*_ = *N*_*Ai*_ x *R*_*i*_ in both models.

3 $${V}_{A}=\left\{\begin{array}{c}\beta ({N}_{{Ai}}\,\times \,{R}_{i}),\hfill	\,{{{{{\rm{if}}}}}}\,{D}_{A} > 0 \hfill \\ \,\,\,\,\,\,{N}_{{Ai}}\,\times \,{R}_{i},\hfill\,	{{{{{\rm{if}}}}}}\,{D}_{A}=0\end{array}\right.$$

4 $${V}_{A}=\left\{\begin{array}{c}\beta {\delta }^{{D}_{A}}({N}_{{Ai}}\,\times \,{R}_{i}),	\,{{{{{\rm{if}}}}}}\,{D}_{A} > 0\hfill\\ \,\,\,\,\,\,\,\,\,\,\,\,\,\,\,{N}_{{Ai}}\,\times \,{R}_{i},\hfill	\,{{{{{\rm{if}}}}}}\,{D}_{A}=0\end{array}\right.$$

We also tested whether time preferences would generalize across reward, effort and punishment intertemporal choices. Bayesian model comparison showed that hyperbolic discounting with category-specific discount rates provided the best trade-off between accuracy and complexity (Supplementary Fig. 1, log group Bayes factor = 212, compared to the second-best model). The balanced accuracy (averaged over participants) was similar for reward, effort and punishment conditions (0.78, 0.79 and 0.77, respectively, see Fig. 2c). Discount rates for reward and effort were weakly correlated (Pearson’s *r*(41) = 0.29, *p* = 0.07) and were on average greater for effort than for reward (paired *t* test, *t*(42) = 2.48, *p* = 0.017). Discount rates estimated for punishment were in between (Fig. 2d), and significantly correlated with both reward (*r*(41) = 0.46; *p* = 0.002) and effort (*r*(41) = 0.61; *p* < 0.001). Participants, therefore, appeared more ‘impulsive’ with effort and more ‘patient’ with reward (i.e., they avoided immediate efforts more than they approached immediate rewards). We checked (Fig. 2e) that this pattern was not dependent on subcategories of reward (food vs. goods, *t*(42) = −0.22, *p* = 0.41), effort (physical vs. mental, *t*(42) = −0.35, *p* = 0.36), or punishment (bodily vs. abstract, *t*(42) = −1.26, *p* = 0.11). Note that the discount rates are comparable here because reward, effort and punishment values were expressed in the same unit (as equivalent gains and losses in euros). The results, therefore, suggest that time preferences for reward and effort can differ both within and between participants.

### Neural correlates of time preferences

**Fig. 3:**
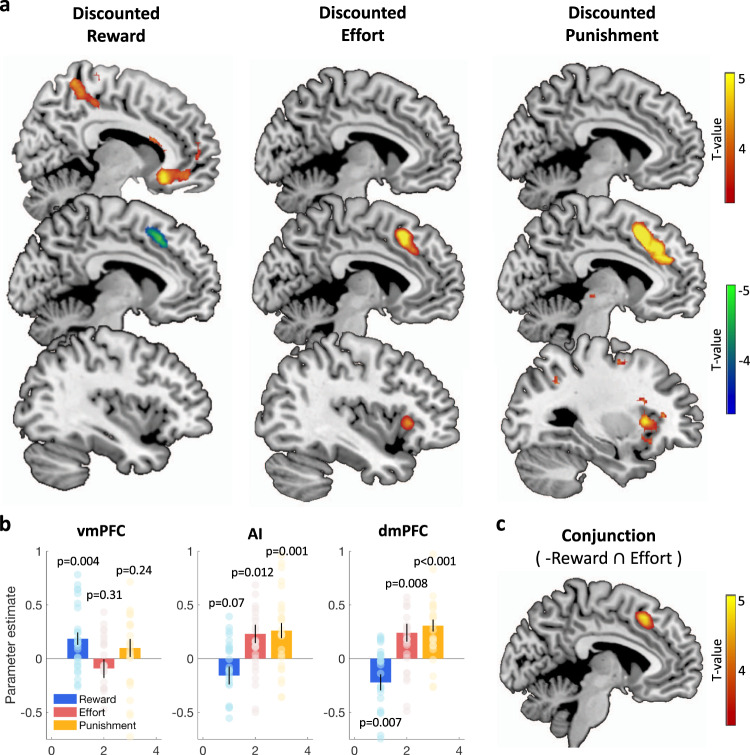
Neural correlates of time preferences. **a** Statistical parametric map (SPM) of discounted reward (chosen minus unchosen option discounted value), discounted effort and discounted punishment (chosen minus unchosen option discounted cost) during intertemporal choice, at the time of deliberation. Positive and negative effects are shown with yellow-red and green-blue color codes. **b** Regression coefficients (betas) of discounted reward (blue), discounted effort (red), and discounted punishment (orange) extracted from anatomically-defined regions of interest (see methods). Error bars are inter-subject standard error of the mean; significance values are based on two-tailed one-sample *t* tests (*n* = 27). Source data are provided as a Source Data file **c** Conjunction between the negative contrast for discounted reward and positive contrast for discounted effort, at the time of deliberation.

We next examined whether these distinct time preferences in the reward and effort domains would involve distinct neural circuits. A standard signature of the choice process in fMRI data is the decision variable, here the difference in value or cost between the two options, at the time of choice. To identify the neural signatures of discounted reward value and discounted effort or punishment cost, we regressed fMRI trial-by-trial time series against the chosen (most appetitive) minus unchosen (least appetitive) reward value, and against the chosen (least aversive) minus unchosen (most aversive) effort or punishment cost. All reward values and effort costs were generated from individual subjective ratings with the best-fitting hyperbolic discounting model (Fig. 3a). We found partially dissociable brain systems, with activity in the ventromedial prefrontal cortex (vmPFC) positively related to discounted reward, activity in the anterior insula (AI) positively related to discounted effort, and activity in the dorsomedial prefrontal cortex (dmPFC) showing an effect in the two domains (negative relation to discounted reward and positive relation to discounted effort). The pattern of activity obtained for punishment was very similar to that of effort (with discounted punishment being positively reflected in the AI and dmPFC).

To illustrate how these regions represent values and costs, we extracted their average parameter estimates (Fig. 3b) in corresponding ROIs taken from published probabilistic atlases (see methods). Activity in the vmPFC correlated positively with discounted reward (*t*(26) = 3.20, *p* = 0.004) but was not significantly affected by discounted effort (*t*(26) = −1.04, *p* = 0.31) or discounted punishment (*t*(26) = 1.19, *p* = 0.24). By contrast, activity in the AI correlated positively with discounted effort (*t*(26) = 2.69, *p* = 0.012) and discounted punishment (*t*(26) = 3.67, *p* = 0.001) but tended to be negatively associated with discounted reward (*t*(26) = −1.88, *p* = 0.07). Finally, activity in the dmPFC was negatively correlated with discounted reward (*t*(26) = −2.96, *p* = 0.007) and positively associated with discounted effort (*t*(26) = 2.87, *p* = 0.008) and discounted punishment (*t*(26) = 5.56, *p* < 0.001). We checked, in two control analyses (Supplementary Fig. 3 and 4), that this pattern of activity was robust across the different subcategories of reward (food vs. good), effort (physical vs. mental), and punishment (bodily vs. abstract), and when defining ROIs on the basis of a different probabilistic atlas (Supplementary Fig. 4). In a conjunction analysis (Fig. 3c), we found that the dmPFC was the only region whose activity was significantly related to both discounted reward (negatively) and discounted effort (positively). This is in line with our previous suggestion that the dmPFC might integrate the costs and benefits signaled by regions more sensitive to one or the other^27^. Note that in many studies (including ours), this fMRI activation cluster has been labeled as dACC (for dorsal Anterior Cingulate Cortex), although strictly speaking it is not located within the cingulate gyrus (but in a more dorsal region of the medial wall, overlapping with the paracingulate gyrus). Our results are consistent with dmPFC/dACC and AI activity being greater when the chosen alternative (least aversive cost) was preferred (to the most aversive cost) by a smaller margin.

### Procrastination behavior

**Fig. 4:**
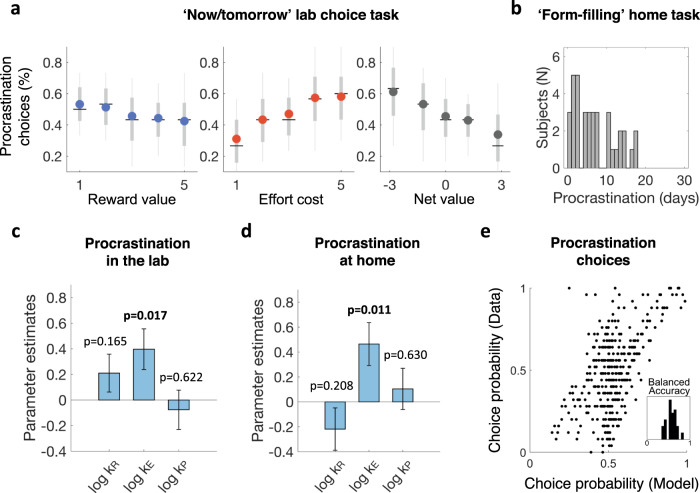
Explaining procrastination with time preferences. **a** Procrastination behavior in the lab ‘now/tomorrow’ choice task. Plots show choice rates for the ‘tomorrow’ option as a function of reward value (left), effort cost (middle), or the task net value (right). On each dot plot, the color mark indicates the mean, the horizonal line indicates the median, the thick whiskers indicate the range from 25th to 75th percentiles, and the thin whiskers indicate the range from 5th to 95th percentiles. (*n* = 43). Source data are provided as a Source Data file **b** Procrastination behavior in the at-home ‘form-filling’ task. Histograms show the distribution of the delay before participants completed and returned administrative forms. **c** Regression estimates of the temporal discount rates for reward (*k*_*R*_), effort (*k*_*E*_) and punishment (*k*_*P*_) obtained from fitting a linear model (also including age and gender) to procrastination level in the ‘now/tomorrow’ choice task. Error bars represent SD; significance values are based on two-tailed one-sample *t* tests (*n* = 43). **d** Regression estimates of reward, effort and punishment discount rates obtained from fitting a linear model (also including age and gender) to procrastination level in the ‘form-filling’ home task. Error bars represent SD; significance values are based on two-tailed one-sample *t* tests (*n* = 37). Note that in both cases (in lab or at home), discount rates are inferred from intertemporal choices, which were observed independently from the procrastination behavior that they contribute to explain. **e** Accuracy of model fitting. The plot shows the correlation between modeled and observed choice frequency for the ‘tomorrow’ option. Individual choices were divided into 8 bins of increasing modeled frequency; each dot represents modeled and observed choice frequency averaged within one bin for one participant. The within-subject (trial-by-trial) fit can be assessed with the distribution of individual balanced accuracy (inset). Balanced accuracy is the average of prediction accuracy calculated separately for the two types of choices (now or later).

The tendency to procrastinate was first assessed in the lab with a task involving choices between performing an effortful task now for an immediate reward, or postponing both task completion and the associated reward until the next day (Fig. 1c). Participants were told that in any case, they would come to the lab twice on two consecutive days, so in practice, they were offered the choice of performing the task proposed in a given trial either during the first current visit or during the second visit on the next day. They were only informed at the end of the first visit that rewards and efforts were fictive and that the second visit would not be implemented. Although this is also a sort of intertemporal choice task, we call it the ‘now/tomorrow’ choice task. The key difference with standard intertemporal choice tasks is that there are two attributes (both reward and effort) to integrate with delay, and not just one (reward or effort). Another difference is that the option proposed at the two delays (today or tomorrow) was the same: it combined a given amount of a reward item and a given amount of an effort item. As expected (Fig. 4a), logistic regression showed that procrastination (preference for ‘tomorrow’) decreased with reward value (*β* = −0.09, *t*(42) = −5.61, *p* < 0.001) and increased with effort cost (*β* = 0.20, *t*(42) = 10.0, *p* < 0.001), hence globally decreased with the net value (*β* = −0.20, *t*(42) = −10.23, *p* < 0.001), i.e. the difference between the discounted benefit and cost associated to the task.

We also assessed procrastination as the delay with which participants completed at home and sent us the 10 administrative forms that were mandatory for receiving their financial compensation (Fig. 1d). The deadline was set to 30 days after the experiment in the lab. Almost all participants procrastinated to some extent (Fig. 4b). Some of them (*n* = 6) never returned the forms and were therefore excluded from the analyses of delay distribution presented hereafter. The delay until completion of administrative forms at home was correlated across participants to the procrastination tendency observed in the lab ‘now/tomorrow’ choice task (*r*(41) = 0.25, *P* = 0.05, one-tailed), and to the procrastination score (*r*(41) = 0.39, *P* = 0.004, one-tailed) on a self-report questionnaire (Lay procrastination scale). However, direct correlation between procrastination in the ‘now/tomorrow’ task and procrastination score on the psychometric scale was not significant (*r*(41) = 0.15, *P* = 0.16, one-tailed). This may suggest that the psychometric score better reflects recurrent procrastination at home than one-shot decision to procrastinate as implemented in the lab choice task.

### Predicting procrastination behavior from time preferences

To test whether procrastination could be explained by the differential temporal discounting of reward and effort, we regressed across participants the level of procrastination observed in the ‘now/tomorrow’ choice task, defined as the selection frequency of ‘tomorrow’ options (Fig. 4c), against temporal discount rates inferred from intertemporal choice tasks. Effort discount rates were significantly associated with procrastination level (*β* = 0.40, *t*(37) = 2.50, *p* = 0.017, two-tailed *t* test, model *R*^2^ = 0.24), whereas the other factors included in the regression model (reward and punishment discount rates plus age and gender) had no significant effect. This pattern was similar when using a different criterion to define procrastinators, i.e. when fitting the regression model to procrastination level defined as the delay taken to send completed administrative forms (Fig. 4d). Again, effort discount rates were significantly associated with procrastination level (*β* = 0.46, *t*(31) = 2.69, *p* = 0.011, two-tailed *t* test, model *R*^2^ = 0.22), while none of the other factors (reward and punishment discount rates plus age and gender) was significant. Thus, the hallmark of procrastination severity, whether measured in the lab or at home, was a steep temporal discounting of effort. This distinctive feature was specific to effort, since other aversive events such as punishments were not more steeply discounted in more severe procrastinators.

We then examined whether differential time preferences for reward and effort could account for individual procrastination behavior in the ‘now/tomorrow’ choice task. The temporal discount rates, as well as subjective ratings of reward and effort items, were rigidly incorporated in the choice model (Eq. 5) explaining the decision to perform the task ‘Now’ or ‘Tomorrow’, without any adjustment to the data (Fig. 4e). This choice model simply compared in a softmax function the net values (i.e., the difference between hyperbolically discounted benefit and cost associated to the proposed task) of the ‘now’ and ‘tomorrow’ options:

5 $${V}_{N\,{{{{{\rm{or}}}}}}\,T}=\,\frac{{N}_{R}\times {R}_{i}}{1+{k}_{R}.D}-\,\frac{{{N}_{E}\times E}_{j}}{1+{k}_{E}.D}$$

With delay D being 0 or 1 depending on the considered option being ‘Now’ or ‘Tomorrow’, *k*_*R*_ and *k*_*E*_ the temporal discount rates for reward and effort (inferred from intertemporal choices), *N*_*R*_ and *N*_*E*_ the quantities of reward i and effort j on offer, whose unitary gain and loss equivalents were *R*_*i*_ and *E*_*j*_ (inferred from individual subjective ratings). The only parameter that was fitted to the now/tomorrow choices was the inverse temperature *θ* of the softmax function, which could not adjust the mean of individual preferences (i.e., procrastination level), but just their stochasticity.

Prediction of trial-by-trial choices was significantly above chance (mean balanced accuracy = 0.57, *t*(42) = 3.88, *p* < 0.001, two-tailed *t* test). We checked that considering different time preferences for reward and effort provided a better fit than using a single common discount rate (Supplementary Fig. 2, log group Bayes factor = 115). This suggests that the steeper discounting of effort relative to reward does help explain the decision to postpone a task until tomorrow. Indeed, it makes postponing a task beneficial because an effort scheduled for tomorrow would appear much less costly, while the reward delayed until tomorrow would not seem much devalued. In other words, the net value of the ‘tomorrow’ option would loom larger than that of the ‘now’ option.

### Predicting procrastination behavior from neural correlates of time preferences

**Fig. 5:**
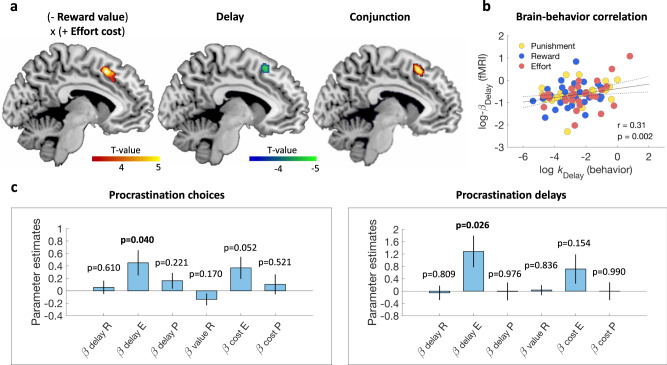
Explaining procrastination with neural correlates of time preferences. **a** Statistical parametric maps (SPM) of delay (chosen minus unchosen delay), – (undiscounted) reward value and + (undiscounted) effort cost, and the conjunction between the two regressors, at the time of deliberation. **b** Correlation between behavioral and neural estimates of the weight on delay (temporal discount rates in choice model versus delay regression estimates in dmPFC activity). Source data are provided as a Source Data file. **c** Regression estimates of beta weights on dmPFC activity for delay and (undiscounted) value or cost of reward, effort, and punishment, obtained from fitting a linear model to procrastination level observed either in the lab choice task (preference for ‘tomorrow’ options) (*n* = 27) or at home (delay in returning administrative forms) (*n* = 23). Note that neural activity was recorded during intertemporal choices, independently from the procrastination behavior assessed in the lab or at home. Error bars represent SD; significance values are based on two-tailed one-sample *t* tests.

To estimate time preferences from their neural correlates, we focused on the dmPFC, which integrated all factors manipulated in the intertemporal choice task. The general linear model (GLM) used to explain choice-related neural activity now included two distinct regressors representing trial-by-trial variations in relative delay and undiscounted decision variable (meaning the difference in reward value or effort cost between the two options). In line with previous results, dmPFC activity was significantly modulated by both factors, as shown by a conjunction analysis (Fig. 5a). We then extracted the delay regression estimates (betas), separately for the reward and effort sessions, within an anatomically-defined dmPFC ROI (see methods). To correct for any subject-specific noise in fMRI data that could corrupt regression estimates, we normalized these betas by the overall activation level (beta weight of the categorical regressor modeling choice onset) in the same ROI. The normalized betas thus represented neural estimates of time preferences, estimated independently from those based on choice behavior (temporal discount rates of the hyperbolic discount model). Yet neural and behavioral measures of time preferences were correlated across participants (Pearson’s *r*(79) = 0.31, *p* = 0.002), combining reward, effort, and punishment estimates (Fig. 5b).

We then tested the link between neural time preferences and procrastination behaviors. We found that procrastination behaviors were associated with neural measures of temporal discount rates in the effort domain only (Fig. 5c). Neural measures of temporal discounting for efforts were significantly associated both with procrastination in the ‘now/tomorrow’ lab choice task (*β* = 0.45, *t*(18) = 2.21, *p* = 0.040, two-tailed *t* test, model *R*^2^ = 0.59) and with the delay to return completed administrative forms (*β* = 1.29, *t*(14) = 2.50, *p* = 0.026, two-tailed *t* test, model *R*^2^ = 0.22). In contrast, there was no significant effect on the neural measures of temporal discount rates for reward or punishment, and no effect of the sensitivity to undiscounted value or cost. We also found no significant effect for gender, and age had a significant effect only on procrastination at home (*β* = −0.55, *t*(14) = −2.20, *p* = 0.044, two-tailed *t* test), but not on procrastination in the lab. Thus, at the neural level, the hallmark of procrastination was a greater temporal discounting of effort expressed in dmPFC activity at the time of choice.

### From static to dynamic computational model of recurrent procrastination

**Fig. 6:**
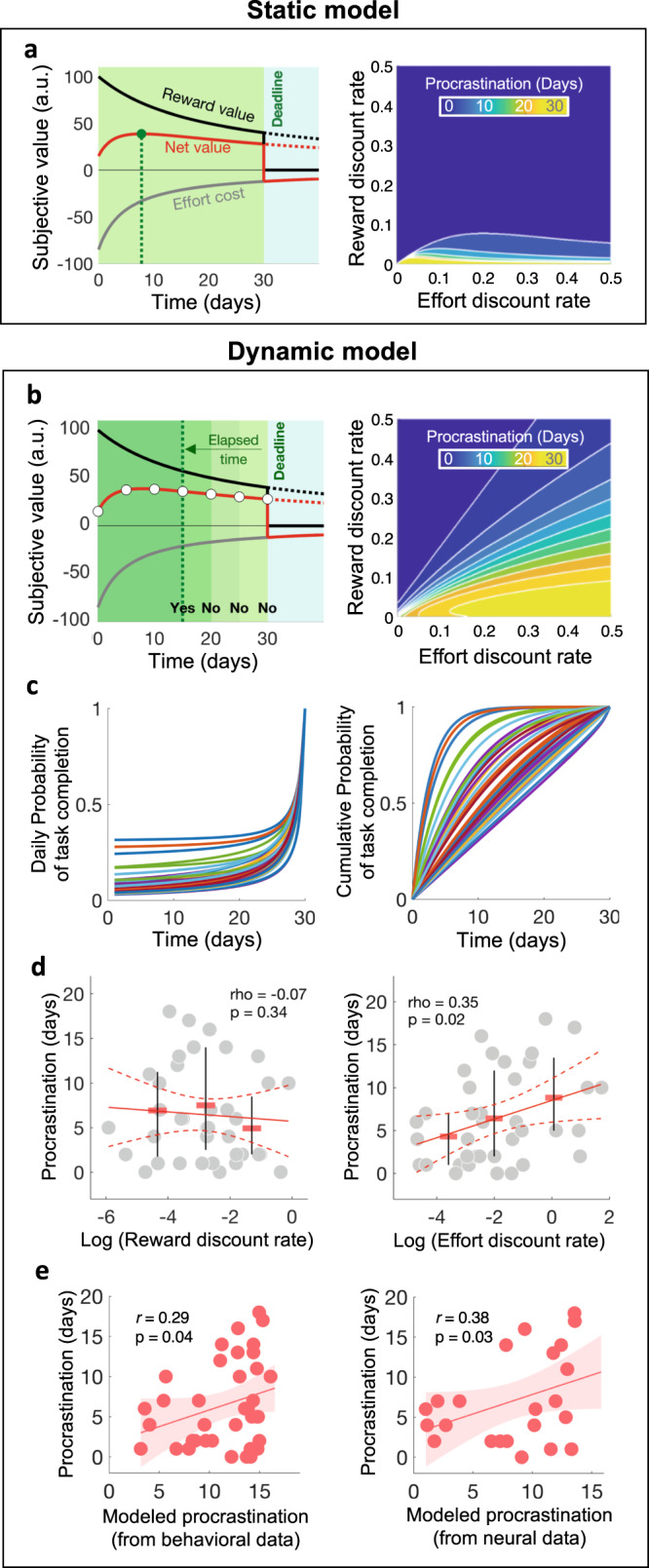
From static to dynamic computational model of procrastination. **a** Static model. Effort cost and reward value are both discounted with time, and reward value vanishes after the deadline. The predicted delay for task completion (i.e., procrastination duration) is the one that maximizes the net value function (green dot). Simulations of procrastination duration under the static model shows that it mostly occurs with low temporal discount rates, even lower for reward than for effort **b** Dynamic model. Every day, the net value of completing the task now is compared to all other remaining available dates (white dots) through a softmax function that provides choice probabilities. As time goes by and the deadline gets closer, the comparison includes fewer dates, which increases the probability of performing the task. This probability obtained for each day is added to the probability of having completed the task on every past day, to generate a cumulative probability. The predicted delay of task completion is the expected value under this cumulative distribution. Simulations of procrastination duration under the dynamic model shows that it occurs with a wide range of temporal discount rates, provided that they are lower for reward than for effort. **c** Daily and cumulative probability of task completion across time, under the dynamic model. Each curve represents one participant. **d** Observed procrastination duration plotted against temporal discount rates for reward (*k*_*R*_) and effort (*k*_*E*_). Each dot is a participant. Red bars represent the means, and whiskers the 25th and 75th percentiles, in three bins of equal size. **e** Inter-participant one-tailed Pearson’s correlation between observed and modeled procrastination duration, under the dynamic model, based on behavioral data or neural data. Each dot is a participant. The number of dots corresponds to the number of participants who did send back the completed forms (*n* = 37 for behavioral data and *n* = 23 for fMRI data). Source data are provided as a Source Data file.

Finally, we developed computational models that articulate unitary decisions to procrastinate, as probed in the ‘now/tomorrow’ choice task, and daily-life recurrent procrastination, as probed in the ‘form-filling’ task. We compared two models that both incorporate the choice model, but fundamentally differed in how procrastination arises. The *static* model implemented a form of pre-commitment, in the sense that the date of task completion was determined a priori, when returning home, as the delay with the highest net value over all possible delays before the deadline (Fig. 6a). The predicted date of task completion (i.e., the duration of procrastination) is, therefore, the one that maximizes the net value function:

6 $${{d}{*}}_{{{{{\rm{task}}}}}}=\,\mathop{{{{{{\rm{argmax}}}}}}}\limits_{d}\left({V}_{d}\right)=\mathop{{{{{{\rm{argmax}}}}}}}\limits_{d}\left(\frac{{R}_{i}}{1+{k}_{R}.d}-\frac{{E}_{i}}{1+{k}_{E}.d}\right)$$

With d*_task_ the optimal day for task completion, *V*_*d*_ the value of performing the task on day d, *R*_*i*_ the financial compensation contingent on completing the administrative forms and *E*_*i*_ the subjective cost of filling in those forms. Note that this model is not necessarily deterministic: the probability of completing the task after a given delay d could be calculated through a softmax function comparing net values over all delays.

In the *dynamic* model, the delay of task completion resulted from iterative decisions, repeated each day, to postpone the task or not (Fig. 6b). Thus, the probability of having completed the task before day *d*_*i*_ is the probability of having not postponed the task on every day before *d*_*i*_, which is given by one minus the product of probabilities to postpone the task (i.e., not doing it now) on every day until *d*_*i*_:

7 $${{{{{\rm{P}}}}}}\left(\tau \le {d}_{i}\right)=1-{\prod }_{d=0}^{{d}_{i}}\,\left(1-{{{{{\rm{P}}}}}}\left({\tau }_{d}=d\right)\right)=\,1-{\prod }_{d=0}^{{d}_{i}}\,\left(1-\frac{{e}^{{\theta V}_{0}}}{{\sum }_{\left(t=0\right)}^{\left(D-d\right)}{e}^{\theta {V}_{t}}}\right)$$

With *τ* the time at which the task is eventually performed, *d*_*i*_ the considered day for task completion, *D* the deadline (30 days), *V*_*t*_ the value of task completion at a given time *t* (*t* = 0 means now), calculated with the same function as in Eq. 6, and *θ* an inverse temperature parameter. Note that the probability to perform the task *P*_*d*_ increases with time because fewer dates before the deadline are available for comparison in the softmax function (Fig. 6c). When deadline is reached (*d* = *D*), this probability becomes one, predicting that the last procrastinators should complete the task then. The cumulative probability of having completed the task *P* is further increasing across days as more probabilistic terms are introduced in the product (see Fig. 6c). Under the dynamic model, the predicted task delay d*_task_ is given by the expected time at which the task is performed:

8 $${{d}{*}}_{{task}}=E\left[\tau \right]={\sum }_{d=0}^{D}d\times P\left(\tau=d\right)={\sum }_{d=1}^{D}d\times \left(P\left(\tau \le d\right)-P\left(\tau \le d-1\right)\right)$$

Simulations showed that under both the static and dynamic models, participants would only postpone the task if temporal discount rates are higher for effort than for reward (Fig. 6a, b), which was the case in our participants. This relates to the net value function that is used in both models. If the temporal discount rate is higher for reward than for effort, the optimal day of task completion is always now, whether the net value is positive or negative (Supplementary Fig. 5). However, only the dynamic model predicts a monotonic relationship between procrastination duration and decreasing reward or increasing effort discount rates. To assess which of these models was more consistent with behavioral data, we first examined how procrastination duration varied as a function of temporal discount rates across participants (Fig. 6d). We found that the tendency to discount effort more steeply was associated with longer procrastination (Pearson’s *r*(35) = 0.35, *p* = 0.02), while the tendency to discount reward more steeply showed an opposite but non-significant effect (Pearson’s *r*(35): −0.07, *p* = 0.34). This pattern is qualitatively consistent with the predictions of the dynamic model and suggests that, although in principle postponing tasks could stem from both decreasing reward and increasing effort temporal discount rate, the latter was driving the procrastination behavior observed in our participants.

We then quantitatively compared the predictions of these models using Bayesian model selection. Note that, again, we did not fit temporal discount rates but rigidly incorporated those inferred from data collected in the intertemporal choice task, together with the subjective cost of administrative form filling expressed by participants in the rating task. Results of Bayesian model selection designated the dynamic model as more plausible than the static model (model log-evidence = −148.7 vs. −169.1, log Bayes Factor = 20.4). Moreover, the dynamic model significantly explained procrastination duration across participants (Fig. 6e; one-tailed Pearson’s *r*(35) = 0.29, *p* = 0.040). Together, these results support the idea that the delay in task completion resulted from iterative decisions to postpone the task, whose probability depended on differential time preferences for effort and reward. Finally, informing the dynamic model with neural time preferences provided equivalent accounts of task completion delays across participants (Fig. 6e; one-tailed Pearson’s *r*(21) = 0.38, *p* = 0.035), which confirms that recurrent procrastination at home may stem from how the brain discounts effort versus reward with time.

## Discussion

The main finding here is that procrastination is related to how effort is discounted with time, relative to reward. Indeed, discount factors estimated during intertemporal decisions between effortful options, whether inferred from behavior or from brain activity, were significantly associated with independent decisions probed in the lab to postpone a rewarded task until the next day. These discount factors were also significantly associated with the delay taken at home to fill in and send back administrative forms, which in turn was significantly correlated with procrastination scores measured using a standard questionnaire (Lay procrastination scale). Because our study may be underpowered to capture the full variability of naturally occurring individual differences in procrastination behavior, these findings require further confirmation in future studies with larger samples. They nevertheless provide initial support to a computational model assuming that (1) unitary decisions to postpone a task until the next day are based on a net value that integrates reward and effort attributes both discounted with time, and that (2) the date of task completion within the allotted time period results from iterative decisions to postpone the task or not. This computational model might therefore account for the recurrent procrastination behavior that is frequently observed in real-life situations.

Regarding unitary decisions to do a task now or tomorrow, we used hyperbolic discounting with time and linear integration of reward and effort. Hyperbolic discounting was used for consistency with intertemporal choices, which were better explained by hyperbolic models than with models including a present bias. This comparison between time discounting functions could not be performed in the now/tomorrow choice task which only compared two delays (0 and 1). Linear integration (without scaling factor) was made possible by using subjective ratings of reward and effort items that provided equivalent gains and losses in euros. In first approximation, the same delays were used for the effort and reward components, since the tasks proposed could be completed in a few seconds or minutes. Thus, the time between task completion and outcome delivery was negligible compared to the delay due to procrastination (one day). Yet, in real-life, task completion can take time and the outcome can be delayed further, which could aggravate procrastination behavior^22^. Our model can be easily generalized to those cases, by using different delays for reward and effort discounting. Thus, the differential time discounting of reward and effort may be considered as a general explanation: it accounts for procrastination behavior even when the aversive task completion is immediately followed by the rewarding outcome (e.g., when passing a phone call to cheer up a relative in pain), and a fortiori when the task is long to complete or the outcome delivered much later (e.g., when preparing an exam to obtain a diploma).

When accounting for now/tomorrow choices within participants, the model suggests that time discounting is not the only factor: procrastination is more likely to occur with less rewarded and more effortful tasks. This is in line with studies that emphasized the failure to regulate emotional responses to task aversiveness as a key determinant of procrastination behavior^6,17,18,22^. However, across participants, these factors were neutralized in the current study by using subjective ratings to adjust the pairing of effort cost and reward value, which may be viewed as emotional responses to task aversiveness and outcome attractivity. Inter-subject variability in procrastination (preference for tomorrow) was mainly captured by the weight on delay in the estimation of effort cost, which may represent a general trait explaining procrastination above and beyond the particular appraisal of specific tasks and outcomes. This weight on delay was obtained independently from fitting choices between effortful options in the intertemporal task, or by estimating the neural response to delay during those choices.

The fact that reward, effort and punishment are differentially discounted with time is supported by our model comparison based on intertemporal choice data. This is not a novel idea: early accounts of intertemporal decisions already suggested that discount rates may vary across attributes of actions and outcomes^29,30^. A specific temporal discount factor for effort was even hypothesized in both the economic^1,28,31^ and motor-control literature^32^. However, although temporal discounting of reward has been studied extensively, very few studies have assessed temporal discounting of effort in humans so far^33,34^, and virtually none in animals. Among aversive outcomes, monetary losses have received more focus. Previous investigations have shown that losses are discounted with functions qualitatively similar to gains but with lower discount rates^29,35–39^, a gain-loss asymmetry called the sign effect^29^. This does not appear to be a general feature of aversive outcomes, since we observed that effort, unlike loss, is more steeply discounted than rewards.

Interestingly, although people generally want to postpone losses for as long as possible, other aversive outcomes, such as receiving a mild electric shock, are sometimes expedited rather than delayed. This negative time preference has been accounted for by dread, i.e. the desire to avoid the experience of anticipating unpleasant future outcomes^29,40,41^. At the neural level, the dread for electric shocks has been related to increased neural activity in the posterior elements of the cortical pain matrix, which has been interpreted as reflecting the attention devoted to the expected physical response^41^. A dread component might also play a role in the effort domain, explaining why tasks such as cleaning cages are rather expedited than delayed when choosing between specific dates^29^. In our pilot study (Exp. 1), which framed delays as the obligation to complete effortful tasks on specific dates, we observed a preference for intermediate delays, consistent with negative discounting of anticipation value combined with positive discounting of consumption value^29^. However, in Exp. 2, where delays were framed as deadlines for completing the effortful task or enduring the punishment, participants showed a preference for longer delays. More than the aversive nature of effort or punishment, it might therefore be their uncontrollable occurrence at precise dates that critically determines the weight of the dread component.

The differential discounting of reward and effort might relate to the recruitment of different brain networks. Neuroimaging studies have implicated both common and separate networks in the temporal discounting of gains and losses^42–44^, but to our best knowledge, no study has ever investigated the neural bases of effort temporal discounting. Our fMRI results are compatible with the view that appetitive and aversive events are signaled by opponent systems (vmPFC and AI, respectively) and integrated in a common region (dmPFC)^26,45–51^. This region was labeled dmPFC here because it was dorsal to the cingulate gyrus, although activation clusters positioned on similar locations are often labeled dACC in the literature on effort, conflict and cognitive control. Despite looking for brain responses to the sequential presentation of the two options, we only found significant neural correlates of option values at the time of choice, possibly because participants waited for this moment to consider the options. In all three clusters of interest, the neural representation of option values was framed by the choice, meaning that brain activity correlated (positively or negatively) with the difference between chosen and unchosen option values, as reported in many previous studies^52–54^. We note that the double dissociation between vmPFC and AI was only partial, as the vmPFC also tended to deactivate with effort (but less reliably than the activation with reward), while the AI also tended to deactivate with reward (but less reliably than the activation with effort). In the dmPFC, the pattern of activity was qualitatively similar to that observed in the AI, but correlations were significant with all option attributes, so the neural response to delay could be extracted from this region for reward, effort and punishment. This extraction of delay regression estimates was independent from the temporal discount factors estimated from choice behavior, but the two markers (neural and behavioral) were correlated across participants. Although the dmPFC was similarly sensitive to effort and punishment on average, procrastination was specifically related to its sensitivity to the delay of effortful tasks, not the delay of punishment outcomes.

This result suggests that procrastination behavior is related to how the brain discounts effort with time, but not necessarily to the specific computation operated by the dmPFC, which could just receive information about option attributes from other brain regions. Also, the direct contribution of the dmPFC to postponing a task could not be assessed here, because we have not scanned participants during the ‘now/tomorrow’ choice task. Still, one may speculate that during this kind of choice, the dmPFC would signal the decision variable, i.e. the net value of doing the task now relative to the net value of doing the task tomorrow (with both effort cost and reward value discounted by one day). Depending on this signal, the brain might engage or not in task completion. Engaging in task completion might itself recruit cognitive control brain systems, with regions such as the lateral prefrontal cortex (lPFC).

Such an interaction between the dmPFC (or dACC) signaling the need for control and the lPFC implementing the required control has already been postulated in published models^55–57^. It could represent a common proximal pathway between procrastination (difficulty in exerting an immediate effort) and impulsivity (difficulty in resisting an immediate pleasure), even if the distal causes are distinct. The existence of both shared and distinct mechanisms may help explain why, although they are distinguishable phenotypic traits^7^, procrastination and impulsivity are correlated across individuals^6,11,58^, and share a significant genetic variability in twin studies^7,59^. Self-regulation failure (or cognitive control deficiency) has indeed been proposed as a common core component of short-sighted behaviors^60,61^. Consistent with this idea, impulsivity has been associated with small lPFC volume^62^, reduced lPFC activity^63^ and lPFC inactivation^64^, while procrastination has been associated with both lPFC volume^9^, lPFC resting-state activity^10^, and control-related lPFC activity^12^. Thus, the tendency to procrastinate might be due to both the dmPFC signaling values in favor of postponing the task and/or the lPFC failing to implement the control necessary for task completion.

Beyond unitary decisions to postpone the task, the results of our model comparison show that recurrent procrastination at home is better explained by iterative decisions (dynamic model) than by a direct readout of the net value function (static model). Without extra-assumptions, the dynamic scheme naturally accounts for the deadline effect (earlier deadline shortening procrastination duration) that has been reported in several studies^24,65,66^. Indeed, at every step, the number of possible time slots remaining to perform the task later is reduced, as the deadline approaches, until the probability of doing the task immediately reaches one on the last allotted slot. Note however that, if no deadline is set, meaning that the number of available time slots is high or even countless, the probability will not vary very much across days, such that the model is likely to make the same decision again and again. If it starts with a low probability of completing the task on the first day, there is a good chance that the task would never be completed, as is also commonly observed in real life.

Compared to the static model, the dynamic model adds a crucial factor in the occurrence of procrastination: the rate at which decisions to complete or postpone the task are considered. As there was no way for us to control the choice rate while participants were back home, we postulated a same constant rate (one decision per day) for everyone. Yet, even if it was the case that some participants thought about filling in the administrative forms every day, it is likely that many of them just forgot about these forms for some time. Integrating the choice rate in the model might therefore help better account for interindividual variability in procrastination behavior. Indeed, the model predicts that individuals who consider completing the task less frequently should procrastinate longer. This feature of the model may account for why prospective memory and external reminders have a significant impact on procrastination:^67–70^ they may increase the rate at which the option of completing the task now is envisaged.

To conclude, our results are consistent with a neuro-computational mechanism accounting for why people repeatedly postpone a task, even when they consider that the benefits surpass the costs (when just comparing reward and effort, ignoring time). Yet these results remain silent about underlying causes, i.e., why effort cost is more discounted with time than reward value in the first place. The explanation might involve attentional processes, if for instance, people focus more on the benefit when the potential task is distant in the future and more on the cost related to its practical implementation when it gets closer in time^71^. At a different time scale, the explanation might involve evolutionary justifications, such as natural selection of the capacity to preserve energetic resources, until it becomes certain that the task needs to be done now. In modern life, procrastination might be adaptive for other reasons, one being that rushing before deadlines might speed up task completion and therefore save time in a busy agenda. In any case, our dynamic model of recurrent procrastination would imply a lack of self-awareness: participants would ignore that by making the same decision again and again, they are likely to miss the optimal date of task completion as defined by their own net value function. Thus, such a cognitive bias might result in many people never completing tasks that would yet improve their well-being.

## Methods

### Participants

In total, 51 healthy adults participated in the study (30 females, median age = 23 ± 2.5 y). This includes three different cohorts (Supplementary Table 1) of participants who participated in a pilot Experiment 1 (*n* = 8), in Experiment 2 with behavioral testing only (*n* = 16), and in Experiment 2 with fMRI (*n* = 27). All participants gave informed consent prior to participating and all data were recorded anonymously. Participants were screened for exclusion criteria: left-handedness, age below 18 or above 40, any history of neurologic or psychiatric illness, regular use of drugs or medication, and contraindications to MRI scanning. Participants were informed that they would receive a fixed amount for their participation (25€ for behavioral studies, 75€ for MRI sessions). The study was approved by the Ethics Committee of the Pitié-Salpêtrière Hospital (Paris, France).

### Tasks

Before performing the tasks, participants were given written instructions, which were also repeated orally step by step. Tasks presentation and behavioral recordings were programmed with MATLAB using the psychophysics Toolbox (www.psychtoolbox.org).

#### Rating tasks

Participants were instructed to report the subjective value of a set of rewards, the subjective cost of a set of efforts, and the subjective cost of a set of punishments, should they (hypothetically) experience these items. For each item, they were asked to indicate the quantity of the item that had the same subjective value (or subjective cost) than earning (or loosing) 1€ and 5€, successively. Items and their units (e.g. number, grams, meters, etc.) were written in the center of the screen, and participants had to indicate the equivalent quantity with a keyboard. Responses were self-paced. The task was made up of one block of reward items equally divided into food items (e.g., pieces of sushi) and goods (e.g., flowers), one block of effort items equally divided into cognitive (e.g., memorizing n digits) and motor efforts (e.g., doing n sit-ups), and one block of punishment items equally divided into bodily (e.g., enduring n mild electric shocks) and abstract losses (e.g., losing my smartphone for n hours). The order of block presentation was counterbalanced across participants. Each block contained 50 items in Exp. 1 and 60 items in Exp. 2.

#### Intertemporal choice task

This task was designed to assess how participants discounted reward, effort, and punishment with time. The punishment condition was used as a control to assess whether effort was discounted by a specific rate or by the same rate as other aversive outcomes. The task was made up of blocks of reward items, blocks of effort items, and blocks of punishment items, whose presentation order was counterbalanced across participants. In each block, participants were presented with a series of hypothetical choices between a sooner/lower quantity of an item and a later/greater quantity of the same item. Delays were randomly drawn from a set of ten delays (0, 1, 2, 3, 5, 7, 10, 14, 21, 30 days). The quantities for each option were adjusted on the basis of item-specific ratings, delays and a priori discount rates to evenly sample the value space. More specifically, the same distribution of option values was used for reward, effort, and punishment, and pseudo-randomly ordered across trials. This intertemporal choice task was used in two separate experiments. While delays indicated the date of delivery for reward items in both experiments, the framing was changed for effort and punishment items in Exp. 2. In Exp. 1 (8 participants), delays indicated the precise date at which efforts had to be exerted, or at which punishments had to be endured, which induced negative time preference (the desire to expedite efforts or suffer punishments sooner). In Exp. 2 (43 participants), however, delays indicated a time limit within which participants could freely decide when to exert the effort, or endure the punishment, which reverted the preferences toward deferring both efforts and punishments. Options were presented on the left and right sides of the screen. The side of the sooner option was counterbalanced across trials. Responses were self-paced, and the selected item was highlighted in red for 500 ms (plus a 0–2000 ms intertrial interval jitter in MRI sessions). The task consisted of 50 choices per block in Exp. 1, and 60 choices per block in Exp. 2, such that no item was repeated within a block. Reward, effort, and punishment blocks were repeated six times in Exp. 1 (behavioral sessions only, 8 participants), four times in behavioral sessions of Exp. 2 (16 participants), and twice in fMRI session of Exp. 2 (27 participants).

#### ‘Now/Tomorrow’ choice task

This task was designed to assess how participants procrastinated in one-shot decisions that involved reward, effort, and delay. On each trial (Fig. 1c), participants of Exp. 2 were proposed offers comprising both a reward and an effort item. They were asked to decide whether to produce the effort “Now” (and get the reward immediately) or “Tomorrow” (and get the reward on the next day). The two responses were randomly displayed on the left and right sides of the bottom part of the screen (position counterbalanced across trials). Reward and effort items were selected randomly from the same sets used in the rating tasks, with no repetition within a block. Reward and effort quantities were adjusted on the basis of participants’ item-specific ratings, such that reward values and efforts costs were drawn from the same uniform distribution and were pseudo-randomly ordered across trials. The task was self-paced and contained 3 blocks of 50 choices. To elicit genuine procrastination choices, participants had been asked, prior to participating in the study, to agree with coming twice to the lab for two experimental sessions on two consecutive days. They were also told that one trial would be randomly selected, and that they would receive the reward on the chosen day (today or tomorrow) and would have to perform the effort on the same day. They were informed only at the end of the experimental session that rewards and efforts were fictive and that a second visit was not required. During debriefing, none of the participants declared having doubted that the second visit would take place and that the rewards and efforts would be actually implemented.

#### Form-filling home task

In order to get an independent and naturalistic measure of recurrent procrastination, participants of exp. 2 (*n* = 43) were informed at the end of the experimental session that they would be given ten printed administrative forms (e.g., passport renewal form; Cerfa documents 10840, 12100, 12485, 12670, 12669, 14445, 14881, 50040, 50239, 50731) at the end of the experimental session, and that they would only receive their financial compensation for participating in the study after filling in the documents and sending a numeric copy by email within a time limit of 30 days. They were told that money transfer would occur as soon as the task was completed, and that no compensation would be transferred after the deadline. In reality, all participants were eventually paid, even those who did not completed the task within the allotted time. However, participants who never sent the forms back (*n* = 6) were not included in the analyses regarding this task, since there was no delay to predict in their case.

### Behavioral analyses

Subjective values (equivalent gains) and subjective costs (equivalent losses) were estimated per unit for each item by averaging ratings made for 1€ questions and ratings made for 5€ questions (divided by 5). Using a linear scaling was a first-order approximation of the true utility function, which we considered reasonable given that reward values and effort costs were within the range of a few euros. To check whether this approximation was indeed reasonable, we fitted linear and power utility functions on individual ratings and use the fitted functions to estimate the values of options presented in the intertemporal choice task. The correlation between values estimated with linear and power functions was 0.98 for reward, 0.97 for effort, and 0.87 for punishment (Pearson’s correlation coefficients). We, therefore, kept the linear approximation to avoid introducing additional flexibility (with power parameters) in the choice models. In all choice tasks, we considered choices as the dependent variables, which were regressed against logistic models including experimental factors: difference in value (or cost) and difference in delay for the intertemporal choice tasks; reward value or effort cost on offer in the now/tomorrow choice task. Procrastination measures in the now/tomorrow choice task and in the form-filling home task were also regressed across participants against behavioral or neural measures of time preferences, as well as age and gender. Two-tailed *t* tests were used to assess the significance of regression coefficients across participants when they could in principle go both ways; one-tailed tests were used when the direction could only be one way (i.e., when testing correlations across participants between alternative measures of the same construct, such as procrastination level). For all *t* tests, the assumption of normality was tested using a Kolmogorov–Smirnov test. Comparisons of parameter means were performed using two-sample *t* tests assuming either equal or unequal variance. The assumption of homoscedasticity was tested using a two-sample *F* test for equal variance.

### Computational models

In intertemporal choice tasks, options were discounted with delay *D* through classical hyperbolic discounting models, in which three discount rates *k*_*R*_, *k*_*E*_ and *k*_*P*_ characterized the steepness of the temporal discounting of reward, effort and punishment, respectively:

1 $${V}_{A}=\,\frac{{N}_{{Ai}}\,\times \,{R}_{i}}{1+k.{D}_{A}}$$

With *V*_*A*_ the value of option A calculated as the number of elements *N*_*A*_ on offer multiplied by the subjective value (*R*_i_, inferred from subjective rating), discounted by the delay *D*_*A*_ multiplied a temporal discount rate k.

We compared this hyperbolic discounting to two classical models of intertemporal choices: a present-bias model (Eq. 3), and a quasi-hyperbolic discounting model (*βδ* model) that includes both a present bias parameter *β* and a constant exponential discount factor *δ* (Eq. 4). Notice that there is a discontinuity in these functions such that, if *D*_*A*_ = 0, *V*_*A*_ = *N*_*Ai*_ × *R*_*i*_ in both models. Further, both *β* and *δ* are constrained so that 0 ≥ *β* ≥ 1 and 0 ≥ δ ≥ 1.

3 $${V}_{A}=\left\{\begin{array}{c}{{{{{\rm{\beta }}}}}}({N}_{{Ai}}\,\times \,{R}_{i}),\,{{{{{\rm{if}}}}}}\,{D}_{A} > 0\\ {N}_{{Ai}}\,\times\,{R}_{i}\,{{{{{\rm{if}}}}}},\,{D}_{A}=0\end{array}\right.$$

4 $${V}_{A}=\left\{\begin{array}{c}{{{{{\rm{\beta }}}}}}{\delta }^{{D}_{A}}({N}_{{Ai}}\,\times \,{R}_{i}),\,{{{{{\rm{if}}}}}}\,{D}_{A} > 0\\ {N}_{{Ai}}\,\times \,{R}_{i},\,{{{{{\rm{if}}}}}}\,{D}_{A}=0\end{array}\right.$$

Decisions were modeled with a softmax function that converted the value difference between the two options *A* and *B* into a choice probability, depending on a temperature parameter *θ* that captured choice stochasticity.

2 $${P}_{A}=\,\frac{1}{1+{e}^{-\theta .({{V}_{A}-V}_{B})}}$$

In the now/tomorrow choice task, the net value of options was modeled as the difference between the reward and the effort on offer, hyperbolically discounted with delay by rates *k*_*R*_ and *k*_*E*_.

5 $${V}_{N\,{{{{{\rm{or}}}}}}\,T}=\,\frac{{N}_{R}\times {R}_{i}}{1+{k}_{R}.D}-\,\frac{{{N}_{E}\times E}_{j}}{1+{k}_{E}.D}$$

With delay D being 0 or 1 depending if the considered option was ‘Now’ or ‘Tomorrow’, *k*_*R*_ and *k*_*E*_ the temporal discount rates for reward and effort (inferred from intertemporal choices), *N*_*R*_ and *N*_*E*_ the quantities of reward *i* and effort *j* on offer, whose unitary gain and loss equivalents were R_i_ and E_j_ (inferred from subjective ratings). Again, a softmax function incorporating an inverse temperature parameter *θ* was used to map net value differences onto choice probabilities. The inverse temperature was individually adjusted, as a free parameter of no interest.

In the form-filling home task, models were meant to predict how many days participants would procrastinate in real-life when required to perform an effortful task before a deadline. We developed two models that both embedded the net value function with differential temporal discounting for effort and reward, but fundamentally differed in how procrastination was generated.

In the *static model*, procrastination results from a maximization, at the initial stage, of the expected net value over the course of the allotted time period. This model predicts that the task is completed on day d*_task_, which corresponds to the delay that maximizes the expected net value.

6 $${{d}{*}}_{{{{{\rm{task}}}}}}=\,\mathop{{{{{{\rm{argmax}}}}}}}\limits_{d}\left({V}_{d}\right)=\mathop{{{{{{\rm{argmax}}}}}}}\limits_{d}\left(\frac{{R}_{i}}{1+{k}_{R}.d}-\frac{{E}_{i}}{1+{k}_{E}.d}\right)$$

With d*_task_ the optimal day for task completion, *V*_*d*_ the value of performing the task on day *d*, *R*_*i*_ the financial compensation contingent on completing the administrative forms and *E*_*i*_ the subjective cost of filling in those forms.

In the dynamic model, by contrast, procrastination arises from binary decisions, repeated over time, about whether to postpone the task or not. On each day *d*, the probability of task completion is given by a softmax function that compares the net value *V*_*0*_ of performing the task now to the net values *V*_*t*_ of performing the task at any other moment before the deadline *D*. Net values were normalized per participant to account for interindividual differences in value range. Let *τ* be the time at which the task is eventually performed. The probability ${{{{{\rm{P}}}}}}\left(\tau \le {d}_{i}\right)$of having completed the task before day *d*_*i*_ is the probability of having not postponed the task on every day before *d*_*i*_, which is given by one minus the product of probabilities to postpone the task (i.e., not doing it now) on every day until *d*_*i*_:

7 $${{{{{\rm{P}}}}}}\left(\tau \le {d}_{i}\right)=1-{\prod }_{d=0}^{{d}_{i}}\,\left(1-{{{{{\rm{P}}}}}}\left({\tau }_{d}=d\right)\right)=\,1-{\prod }_{d=0}^{{d}_{i}}\,\left(1-\frac{{e}^{{\theta V}_{0}}}{{\sum }_{\left(t=0\right)}^{\left(D-d\right)}{e}^{\theta {V}_{t}}}\right)$$

where τ_*t*_ is the delay that would be chosen at time *t*, under the assumption that the task was postponed until time *t*. Note that, by construction, ${{{{{\rm{P}}}}}}\left({\tau }_{D}=D\right)=1$, i.e., the task cannot be postponed beyond the deadline. Under the dynamic model, the predicted task delay d*_task_ is given by the expected time at which the task is performed:

8 $${{d}{*}}_{{{{{\rm{task}}}}}}=E\left[\tau \right]={\sum }_{d=0}^{D}d\times P\left(\tau=d\right)={\sum }_{d=1}^{D}d\times \left({{{{{\rm{P}}}}}}\left(\tau \le d\right)-{{{{{\rm{P}}}}}}\left(\tau \le d-1\right)\right)$$

where we have assumed that the decision about whether the task is postponed or not is repeated on each consecutive day.

To illustrate how the predicted delay of task completion was determined for each model, we simulated the expected net value of an option combining a subjective gain of 100 a.u. and a subjective cost of 85 a.u with a temporal discounting rate of 0.05 for reward and 0.2 for effort. We also performed 10^6^ simulations, varying all free parameters with a deadline set to 30 days, and estimated the average procrastination duration in the space of discount rate parameters, marginalizing over reward values and effort costs. The simulations spanned the following ranges for the different parameters and variables: *Ke* = 0–0.5, *Kr* = 0–0.5, *R* = 40–60 a.u., *E* = 20–40 a.u., *θ* = 0.5. We then informed these models with participant’s data (expected gratification, form-filling cost ratings, and temporal discounting rates for reward and effort) to model the observed procrastination duration.

### Bayesian model estimation and selection

The different models were inverted using a variational Bayes approach under the Laplace approximation^72,73^, implemented in the VBA toolbox (available at https://mbb-team.github.io/VBA-toolbox). This algorithm not only inverts nonlinear models with an efficient and robust parameter estimation, but also estimates the model evidence, which represents a trade-off between accuracy (goodness of fit) and complexity (degrees of freedom). The following non informative priors were used for parameters estimation: *μ* = 0, *σ* = 1 for *Kr* and *Ke*; *μ* = 1, *σ* = 1 for *θ*. The model log-evidence was then used as a criterion to select which model best accounted for temporal discounting and recurrent procrastination.

### Neuroimaging acquisition

Multiband T2*-weighted echoplanar images (EPIs) were acquired with blood oxygen level-dependent (BOLD) contrast on a 3.0 T MRI scanner (Siemens Trio) in 27 participants. The sample size was chosen to be larger than the sample size used in a previous study from our group that identified option values signals during intertemporal choice^74^. A tilted plane acquisition sequence was used to optimize functional sensitivity in the orbitofrontal cortex. To cover the whole brain (except the cerebellum), we used the following parameters: 1022 ms repetition time (TR), 25 ms echo time (TE), 45 slices, 2.5 mm slice thickness, 0.5 mm interslice gap, 2.5 mm × 2.5 mm in-plane resolution, 80 × 80 matrix, 60° flip angle, x3 acceleration factor. T1-weighted structural images were also acquired, coregistered with the mean EPI, segmented and normalized to a standard T1 template, and averaged across all participants to allow group-level anatomical localization. EPIs were analyzed in an event-related manner, within a GLM, using SPM12 (www.fil.ion.ucl.ac.uk/spm). The first five volumes of each session were discarded to allow for T1 equilibration effects. Preprocessing consisted of spatial realignment, normalization using the same transformation as structural images, and spatial smoothing using a Gaussian kernel with a full-width at half-maximum (FWHM) of 8 mm.

### Neuroimaging analysis

We used a first GLM to generate SPMs of discounted reward and effort, as follows. All trials of the intertemporal choice tasks were modeled as single events with Dirac delta-functions at the time of deliberation onset. The difference in discounted value between chosen and unchosen rewards, or the difference in discounted cost between chosen and unchosen efforts or punishments, was incorporated as parametric modulation. These decision variables are typically signaled during the comparison of options by neural activity in key brain regions involved in value or cost estimation^52–54^. All regressors of interest were convolved with a canonical hemodynamic response function. To correct for motion artifact, subject-specific realignment parameters were modeled as covariates of no interest. Linear contrasts of regression coefficients (betas) were computed at the individual participant level and then taken to a group-level random effect analysis (using one-sample *t* test). All reported significant activations contained voxels surviving a threshold of *p* < 0.05 after familywise error correction for multiple comparisons at the cluster level (c-FWE), unless otherwise mentioned.

To specify how procrastination was related to reward and effort temporal discounting, we extracted betas from a second GLM that incorporated one event per trial, at the time of deliberation onset. The difference in delay and in undiscounted value or cost between chosen and unchosen options were incorporated as parametric modulation. We extracted betas from vmPFC, AI and dmPFC regions of interest (ROIs) defined from published atlases: the vmPFC ROI corresponded to the 14 m area of the Mackey and Petrides probabilistic atlas^75^; the anterior insula ROI consisted of the anterior short gyrus and the anterior inferior cortex from the Hammersmith atlas^76^; and the dmPFC ROI corresponded to the paracingulate regions of the Harvard-Oxford brain atlas distributed with FSL (https://www.fmrib.ox.ac.uk/fsl) that do not extend anterior to the genu of the corpus callosum. Procrastination measures in the lab and at home were then regressed against a linear model that included the betas obtained for delay, undiscounted value and cost, as well as age and gender.

### Reporting summary

Further information on research design is available in the Nature Research Reporting Summary linked to this article.

## Supplementary information

Supplementary Information

Reporting Summary

## Data Availability

The raw behavioral data that support the findings of this study and brain maps are available for download (https://github.com/rlebouc/procrastination). Raw fMRI data can be obtained from the corresponding author upon reasonable request. Source data are provided with this paper.

## Source data

Source Data

## Code availability

All computer codes for analysis will be made available by the corresponding author upon reasonable request.
