# Publisher Correction: A neuro-computational account of procrastination behavior

**DOI:** 10.1038/s41467-022-34142-7

**Published:** 2022-10-21

**Authors:** Raphaël Le Bouc, Mathias Pessiglione

**Affiliations:** 1 Motivation, Brain and Behavior (MBB) Lab, Paris Brain Institute (ICM), Sorbonne University, Inserm, CNRS, Pitié-Salpêtrière hospital, Paris, France; 2 Department of Neurology, Pitié-Salpêtrière hospital, Sorbonne University, Assistance Publique – Hôpitaux de Paris (APHP), Paris, France

**Keywords:** Decision, Reward, Motivation, Computational neuroscience, Human behaviour

Correction to: *Nature Communications* 10.1038/s41467-022-33119-w, published online 26 September 2022

This Article erroneously swapped the first and last names of the authors Raphaël Le Bouc and Mathias Pessiglione, which were incorrectly given as Le Bouc Raphaël and Pessiglione Mathias. The error has been corrected in the PDF or HTML versions of the Article.

