# Supplementary material for: A neuro-computational account of procrastination behavior
Source: Nat Commun. 2022 Sep 26;13:5639. doi: 10.1038/s41467-022-33119-w (PMC9513091; doi:10.1038/s41467-022-33119-w)
Supplement: Supplementary file 1 — Supplementary Information [file 41467_2022_33119_MOESM1_ESM.pdf]

Supplementary Information

Supplementary Table 1. Demographic details.

	Cohorts				
	**Cohort A** Experiment 1 (behavioral testing)	**Cohort B** Experiment 2 (behavioral testing)	**Cohort C** Experiment 2 (fMRI testing)	All cohorts combined	*p*
Number	8	16	27	51	
Female/Male	4/4	10/6	16/11	30/21	all $p > 0.05$
Age (median ± sd)	24 ± 5.3	22 ± 3.1	23 ± 2.1	23 ± 2.5	all $p > 0.05$
Lay Procrastination scale (score)	–	51 ± 11.8	55.5 ± 15.0	53.9 ± 13.9	all $p > 0.05$

No significant pairwise difference was observed between cohorts, using either Chi-2 test or two-sample t-test for binary and continuous dependent variables.

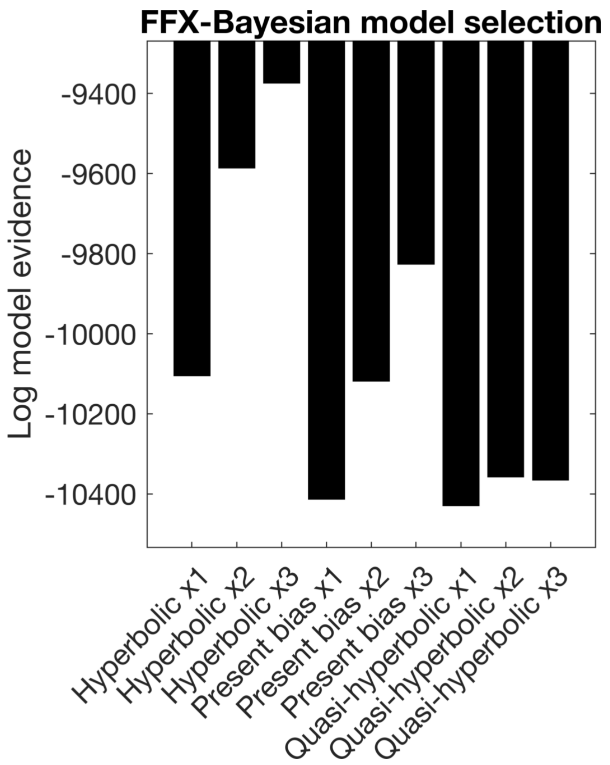

Supplementary Figure 1. Bayesian model selection of the temporal discounting function used to fit intertemporal choices. The plausibility of the different functions was estimated on the basis of their log-model evidence in a fixed-effect analysis. Models differed in the shape of temporal discounting (hyperbolic function, present bias, quasi-hyperbolic function) and in the number of discount rate parameters (x1: one common discount rate for all categories; x2: two discount rates for reward on the one hand, effort and punishment on the other hand; x3: three discount rates for reward, effort and punishment). The smallest negative log-evidence designates the most plausible model (hyperbolic x3).

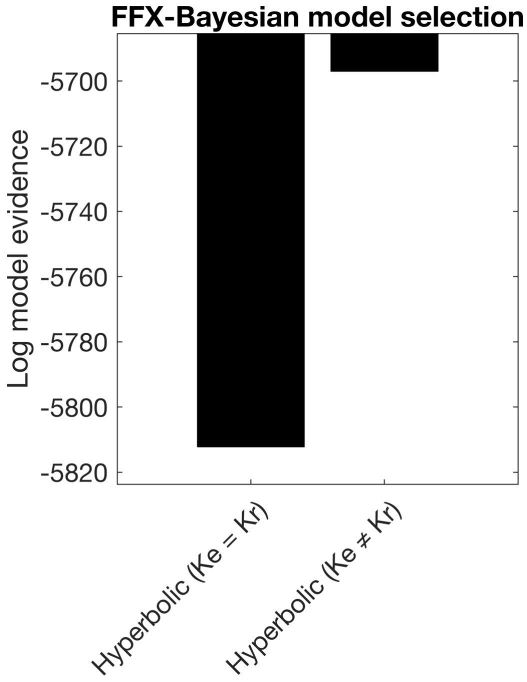

Supplementary Figure 2. Bayesian model selection of the temporal discounting function used to fit 'Now/Tomorrow' choices. The plausibility of the different functions was estimated on the basis of their log-model evidence in a fixed-effect analysis. Models differed in the number of discount rate parameters (*Ke* = *Kr*: one common discount rate; *Ke* ≠ *Kr*: two different discount rates for reward and effort). The smallest negative log-evidence designates the most plausible model (*Ke* ≠ *Kr*).

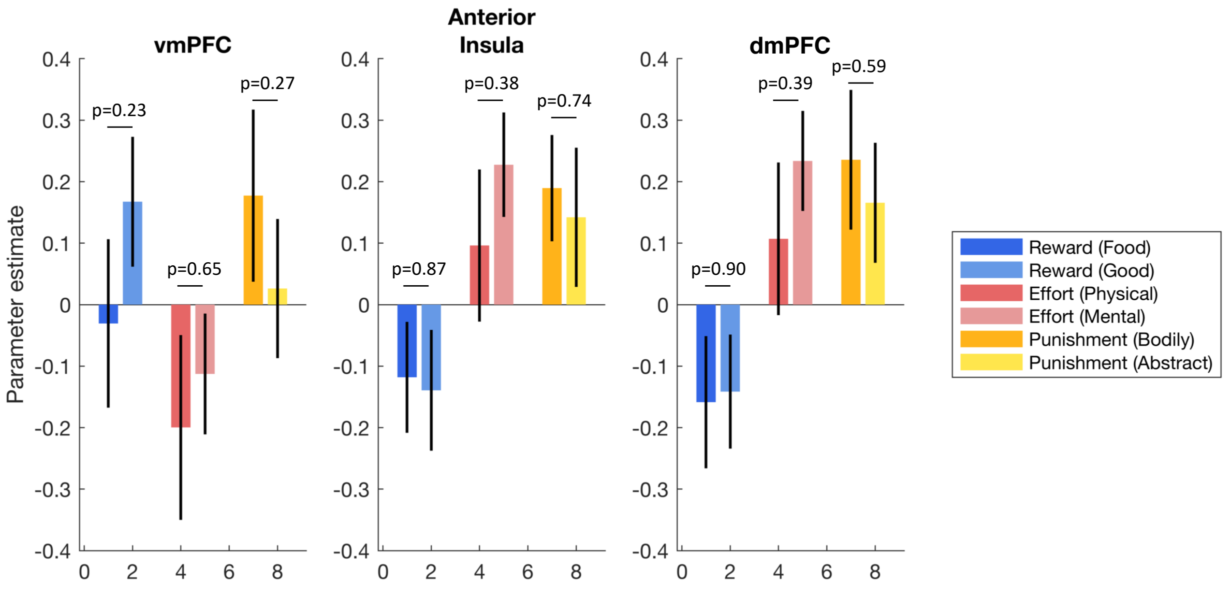

Supplementary Figure 3. Neural correlates of time preferences across subcategories of items. Regression estimates (betas) of discounted reward value (food and good, dark and light blue), discounted effort cost (physical and mental, dark and light red), and discounted punishment cost (bodily and abstract, orange and yellow). No significant difference was found between the two subcategories of reward, effort and punishment domains. Regions of interest are the same as in the main text (Fig. 3). Error bars are inter-subject standard error of the mean; significance values are based on two-tailed paired t-tests (n = 27). Source data are provided as a Source Data file.

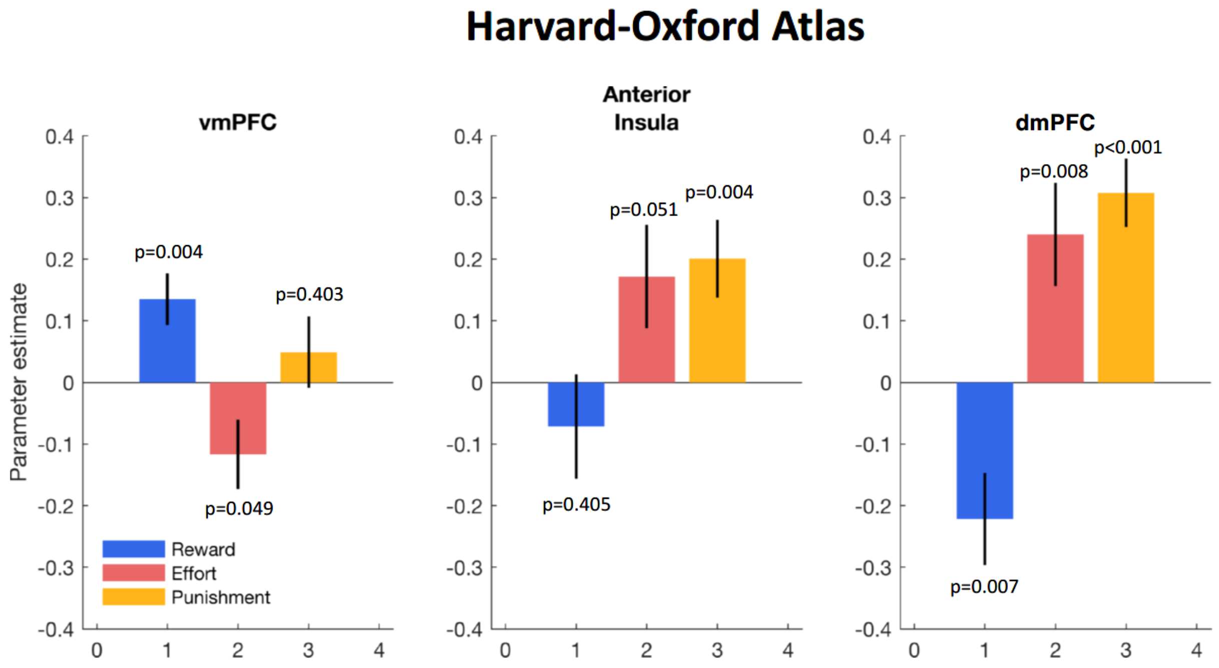

Supplementary Figure 4. Neural correlates of time preferences extracted from a different probabilistic atlas. Regression estimates (betas) of discounted reward value (blue), discounted effort cost (red), and discounted punishment cost (orange) extracted from anatomically-defined regions of interest taken from the Harvard-Oxford (HO) atlas distributed with the FSL software package [1]. The vmPFC ROI corresponds to the 'frontal medial cortex', the dmPFC ROI corresponds to the 'paracingulate gyrus', the AI ROI corresponds to the part of the insular cortex that is covered by the inferior frontal cortex (following [2]). Error bars are inter-subject standard error of the mean; significance values are based on two-tailed one-sample t-tests (*n* = 27). Source data are provided as a Source Data file. vmPFC, ventromedial prefrontal cortex; dmPFC, dorsomedial prefrontal cortex.

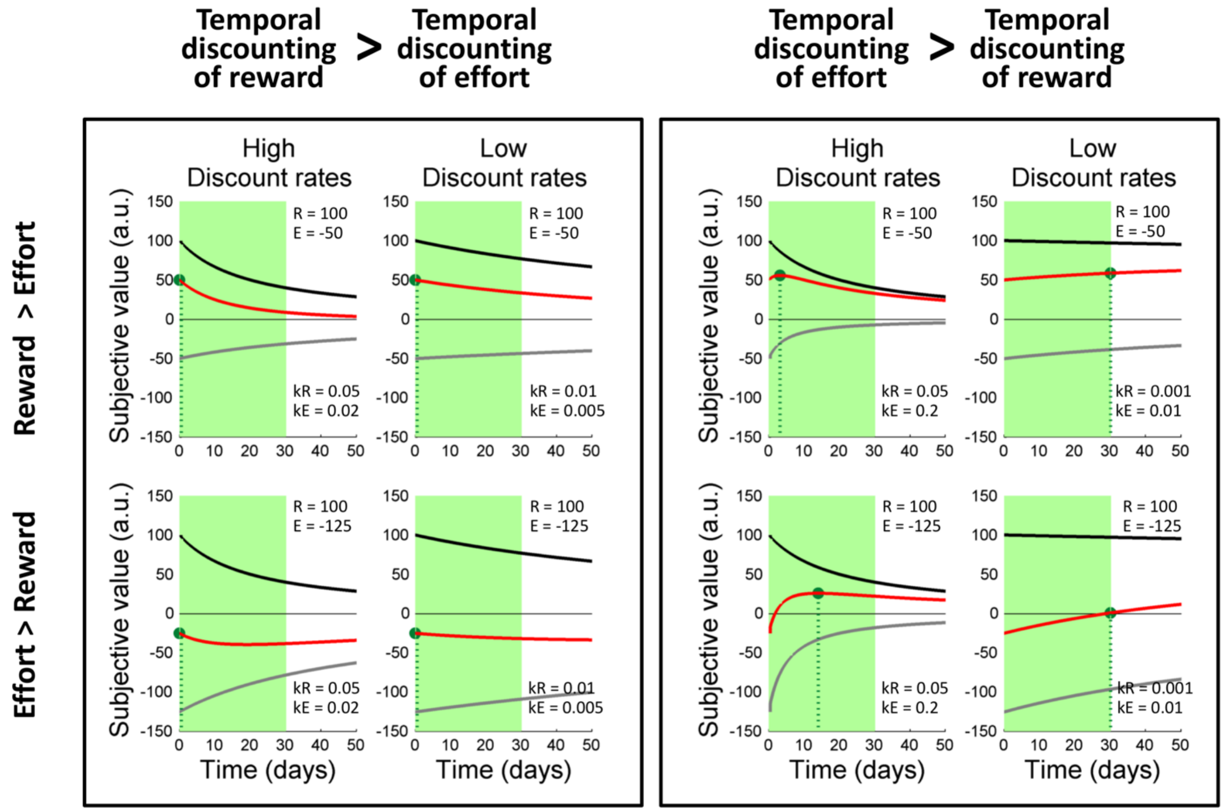

Supplementary Figure 5. Simulation of the optimal task completion date according to the static model of procrastination. The plots display the discounted reward value (black), the discounted effort cost (grey), and their difference, i.e. the net value function (red), across time. **(Left panel)** Simulations with a higher temporal discount rate for reward k_R than for effort k_E. **(Right panel)** Simulations with a higher temporal discount rate for effort k_E than for reward k_R. Each panel also shows the effect of high versus low discount rates. **(Top row)** Simulations with reward value *R* exceeding effort cost *E*. **(Bottom row)** Simulations with effort cost *E* exceeding reward value *R*. Green dots indicate the maximum of the net value function within the allotted time window (light green rectangles) for returning completed administrative forms. a.u., arbitrary units.

Supplementary References

1 The FSL software package is available at: http://www.fmrib.ox.ac.uk/fsl/

2 Faillenot, I., Heckemann, R. A., Frot, M. & Hammers, A. Macroanatomy and 3D probabilistic atlas of the human insula. *Neuroimage* **150**, 88-98, doi:10.1016/j.neuroimage.2017.01.073 (2017).
