# Supplementary material for: A neuro-computational account of procrastination behavior
Source: Nat Commun. 2022 Sep 26;13:5639. doi: 10.1038/s41467-022-33119-w (PMC9513091; doi:10.1038/s41467-022-33119-w)
Supplement: Supplementary file 2 — Reporting Summary [file 41467_2022_33119_MOESM2_ESM.pdf]

nature research

Corresponding author(s): Le Bouc Raphaël

Last updated by author(s): Aug 10, 2022

Reporting Summary

Nature Research wishes to improve the reproducibility of the work that we publish. This form provides structure for consistency and transparency in reporting. For further information on Nature Research policies, see our Editorial Policies and the Editorial Policy Checklist.

Statistics

For all statistical analyses, confirm that the following items are present in the figure legend, table legend, main text, or Methods section.

n/a	Confirmed	
☐	☒	The exact sample size (*n*) for each experimental group/condition, given as a discrete number and unit of measurement
☐	☒	A statement on whether measurements were taken from distinct samples or whether the same sample was measured repeatedly
☐	☒	The statistical test(s) used AND whether they are one- or two-sided<br>*Only common tests should be described solely by name; describe more complex techniques in the Methods section.*
☐	☒	A description of all covariates tested
☐	☒	A description of any assumptions or corrections, such as tests of normality and adjustment for multiple comparisons
☐	☒	A full description of the statistical parameters including central tendency (e.g. means) or other basic estimates (e.g. regression coefficient) AND variation (e.g. standard deviation) or associated estimates of uncertainty (e.g. confidence intervals)
☐	☒	For null hypothesis testing, the test statistic (e.g. *F*, *t*, *r*) with confidence intervals, effect sizes, degrees of freedom and *P* value noted<br>*Give P values as exact values whenever suitable.*
☐	☒	For Bayesian analysis, information on the choice of priors and Markov chain Monte Carlo settings
☒	☐	For hierarchical and complex designs, identification of the appropriate level for tests and full reporting of outcomes
☐	☒	Estimates of effect sizes (e.g. Cohen's *d*, Pearson's *r*), indicating how they were calculated

Our web collection on statistics for biologists contains articles on many of the points above.

Software and code

Policy information about availability of computer code

Data collection	PsychToolbox (Version 3) was employed for behavioral data collection at the laboratory and for stimulus delivery inside the MRI scanner.<br>A 3.0T Siemens Trio scanner was used for the collection of MRI data.<br>Matlab (Version 2019a) by MathWorks<br>SPM toolbox (Version 12.0)<br>VBA toolbox for Matlab (available at https://mbb-team.github.io/VBA-toolbox)
Data analysis	All computer codes used during the current study are available from the corresponding author upon reasonable request.

For manuscripts utilizing custom algorithms or software that are central to the research but not yet described in published literature, software must be made available to editors and reviewers. We strongly encourage code deposition in a community repository (e.g. GitHub). See the Nature Research guidelines for submitting code & software for further information.

Data

Policy information about availability of data

All manuscripts must include a data availability statement. This statement should provide the following information, where applicable:

- Accession codes, unique identifiers, or web links for publicly available datasets
- A list of figures that have associated raw data
- A description of any restrictions on data availability

The data that support the findings of this study and brain maps are available for download (https://github.com/rlebouc/procrastination).
Source data are provided with this paper.

Field-specific reporting

Please select the one below that is the best fit for your research. If you are not sure, read the appropriate sections before making your selection.

☐ Life sciences ☒ Behavioural & social sciences ☐ Ecological, evolutionary & environmental sciences

For a reference copy of the document with all sections, see nature.com/documents/nr-reporting-summary-flat.pdf

Behavioural & social sciences study design

All studies must disclose on these points even when the disclosure is negative.

Study description	Behavioral and fMRI study with quantitative data.<br>Three different cohorts of subjects participated in a pilot Experiment 1 (n = 8), in Experiment 2 with behavioral testing only (n = 16), and in Experiment 2 with fMRI (n = 27).
Research sample	51 healthy adults participated in the study (30 females, median age = 23 ± 2.5 y). This sample is not representative of the general population. The sample size was chosen to be larger than the sample size used in a previous study from our group that identified option values signals during inter-temporal choice (Lebreton et al., 2009).
Sampling strategy	For the fMRI study, the sample size was chosen to be larger than the sample size used in a previous study from our group that identified option values signals during inter-temporal choice (Lebreton et al., 2009).<br>For participants who participated in the behavioural testing only, no sample size calculation was performed since behavioural analyses were performed combining participants from the fMRI and the behavioral study.
Data collection	Tasks presentation and behavioral recordings were programmed with MATLAB using the psychophysics Toolbox (www.psychtoolbox.org).<br>No one was present besides the participant and the researcher. The experimenter was not blind to the study hypothesis.
Timing	Data collection was performed between 21/08/2013 and 01/01/2014.
Data exclusions	In the Form-filling home task, participants who never sent the forms back (n = 6) were not included in the analyses regarding this task, since there was no delay to predict in their case.
Non-participation	No participant dropped out/declined participation.
Randomization	Randomization was not applicable in this study since all participants performed the same cognitive task.

Reporting for specific materials, systems and methods

We require information from authors about some types of materials, experimental systems and methods used in many studies. Here, indicate whether each material, system or method listed is relevant to your study. If you are not sure if a list item applies to your research, read the appropriate section before selecting a response.

Materials & experimental systems

n/a	Involved in the study	
☒	☐	Antibodies
☒	☐	Eukaryotic cell lines
☒	☐	Palaeontology and archaeology
☒	☐	Animals and other organisms
☐	☒	Human research participants
☒	☐	Clinical data
☒	☐	Dual use research of concern

Methods

n/a	Involved in the study	
☒	☐	ChIP-seq
☒	☐	Flow cytometry
☐	☒	MRI-based neuroimaging

Human research participants

Policy information about studies involving human research participants

Population characteristics	See above
Recruitment	Participants were recruited from the community, and were screened for exclusion criteria: left-handedness, age below 18 or above 40, any history of neurologic or psychiatric illness, regular use of drugs or medication, and contraindications to MRI scanning.<br>All participants were recruited from the undergraduate and postgraduate population of Paris Universities via advertisements posted on the online recruitment system of the French National Center for Scientific Research (CNRS) available at www.risc.cnrs.fr. All volunteers received monetary compensation for their participation in line with our institution policies. There may be a selection bias given that participants who agreed to take part in our experiments are generally young adults and are enrolled in higher education. Although, we do not expect this to have a significant influence on our results, future studies with more representative samples of the population will be required to assess the generalisability of our findings.
Ethics oversight	The study was approved by the Ethics Committee of the Pitié-Salpêtrière Hospital (Paris, France).

Note that full information on the approval of the study protocol must also be provided in the manuscript.

Magnetic resonance imaging

Experimental design

Design type	Event-related
Design specifications	The task consisted of 60 choices per block. Reward, effort, and punishment blocks were repeated twice in fMRI session. Responses were self-paced, and were followed by a 0-2000ms intertrial interval jitter.
Behavioral performance measures	In the intertemporal choice tasks, we considered choices as the dependent variables, which were regressed against logistic models including experimental factors: difference in value (or cost) and difference in delay.

Acquisition

Imaging type(s)	functional
Field strength	3T
Sequence & imaging parameters	Multiband T2*-weighted echoplanar images (EPIs) were acquired with blood oxygen level-dependent (BOLD) contrast on a 3.0T MRI scanner (Siemens Trio). To cover the whole brain (except the cerebellum), we used the following parameters: 1022ms repetition time (TR), 25ms echo time (TE), 45 slices, 2.5 mm slice thickness, 0.5 mm interslice gap, 2.5 mm x 2.5 mm in-plane resolution, 80x80 matrix, 60° flip angle, x3 acceleration factor.
Area of acquisition	The area of acquisition included the whole brain except the cerebellum

Diffusion MRI ☐ Used ☒ Not used

Preprocessing

Preprocessing software	SPM12 was used for preprocessing fMRI data.<br>Preprocessing consisted of spatial realignment, normalization using the same transformation as structural images, and spatial smoothing using a Gaussian kernel with a full-width at half-maximum (FWHM) of 8 mm.
Normalization	T1-weighted structural images were also acquired, coregistered with the mean EPI, segmented and normalized to a standard T1 template, and averaged across all participants to allow group-level anatomical localization.
Normalization template	SPM standard T1 template
Noise and artifact removal	To correct for motion artifact, subject-specific realignment parameters were modeled as covariates of no interest.
Volume censoring	n/a

Statistical modeling & inference

Model type and settings	We used a first GLM to generate SPMs of discounted reward and effort, as follows. All trials of the intertemporal choice tasks were modelled as single events with Dirac delta-functions at the time of deliberation onset. The difference in discounted value between chosen and unchosen rewards, or the difference in discounted cost between chosen and unchosen efforts or punishments, was incorporated as parametric modulation.

To specify how procrastination was related to reward and effort temporal discounting, we extracted betas from a second GLM that incorporated one event per trial, at the time of deliberation onset. The difference in delay and in undiscounted value or cost between chosen and unchosen options were incorporated as parametric modulation.

All regressors of interest were convolved with a canonical hemodynamic response function. Linear contrasts of regression coefficients (betas) were computed at the individual subject level and then taken to a group-level random effect analysis (using one-sample t-test).

Effect(s) tested

In the first GLM, we tested the effect of discounted reward value and discounted effort or punishment cost.
In the second GLM, we tested the effect of delay and undiscounted reward value or effort cost.

Specify type of analysis: ☐ Whole brain ☐ ROI-based ☒ Both

Anatomical location(s): anatomical locations were based on probabillistic atlases.

Statistic type for inference
(See Eklund et al. 2016)

cluster level (c-FWE) correction

Correction

All reported significant activations contained voxels surviving a threshold of $p < 0.05$ after familywise error correction for multiple comparisons at the cluster level (c-FWE)

Models & analysis

n/a	Involved in the study
☒	☐ Functional and/or effective connectivity
☒	☐ Graph analysis
☒	☐ Multivariate modeling or predictive analysis
